# Exercise and fatigue: integrating the role of K^+^, Na^+^ and Cl^−^ in the regulation of sarcolemmal excitability of skeletal muscle

**DOI:** 10.1007/s00421-023-05270-9

**Published:** 2023-08-16

**Authors:** Jean-Marc Renaud, Niels Ørtenblad, Michael J. McKenna, Kristian Overgaard

**Affiliations:** 1 Faculty of Medicine, Department of Cellular and Molecular Medicine, University of Ottawa, 451 Smyth Rd., Ottawa, ON K1H 8M5 Canada; 2 Department of Sports Science and Clinical Biomechanics, University of Southern Denmark, Odense, Denmark; 3 Institute for Health and Sport, Victoria University, Melbourne, VIC 8001 Australia; 4 College of Physical Education, Southwest University, Chongqing, China; 5 College of Sport Science, Zhuhai College of Science and Technology, Zhuhai, China; 6 Exercise Biology, Department of Public Health, Aarhus University, Aarhus, Denmark

**Keywords:** ClC-1 channel, K_ATP_ channel, Membrane excitability, Force potentiation, Force depression, Metabolic stress

## Abstract

Perturbations in K^+^ have long been considered a key factor in skeletal muscle fatigue. However, the exercise-induced changes in K^+^ intra-to-extracellular gradient is by itself insufficiently large to be a major cause for the force decrease during fatigue unless combined to other ion gradient changes such as for Na^+^. Whilst several studies described K^+^-induced force depression at high extracellular [K^+^] ([K^+^]_e_), others reported that small increases in [K^+^]_e_ induced potentiation during submaximal activation frequencies, a finding that has mostly been ignored. There is evidence for decreased Cl^−^ ClC-1 channel activity at muscle activity onset, which may limit K^+^-induced force depression, and large increases in ClC-1 channel activity during metabolic stress that may enhance K^+^ induced force depression. The ATP-sensitive K^+^ channel (K_ATP_ channel) is also activated during metabolic stress to lower sarcolemmal excitability. Taking into account all these findings, we propose a revised concept in which K^+^ has two physiological roles: (1) K^+^-induced potentiation and (2) K^+^-induced force depression. During low-moderate intensity muscle contractions, the K^+^-induced force depression associated with increased [K^+^]_e_ is prevented by concomitant decreased ClC-1 channel activity, allowing K^+^-induced potentiation of sub-maximal tetanic contractions to dominate, thereby optimizing muscle performance. When ATP demand exceeds supply, creating metabolic stress, both K_ATP_ and ClC-1 channels are activated. K_ATP_ channels contribute to force reductions by lowering sarcolemmal generation of action potentials, whilst ClC-1 channel enhances the force-depressing effects of K^+^, thereby triggering fatigue. The ultimate function of these changes is to preserve the remaining ATP to prevent damaging ATP depletion.

## Introduction: the initial concept about K^+^ and fatigue

Muscle fatigue is defined as a transient decrease in the capacity of skeletal muscles to generate force or do work when repetitively activated. Fenn and colleagues in the 1930’s documented K^+^ loss, Na^+^ and water gain in contracting skeletal muscle (Fenn 1938, 1937; Fenn and Cobb 1936). A very large number of reports that followed these studies were on muscle contraction-induced changes in plasma, muscle interstitial and intracellular [K^+^] (see the extensive companion review by McKenna et al. (2023). Although the mechanism for the K^+^ loss was not understood at the time, Fenn proposed that a progressive K^+^ loss could cause the simultaneously observed loss of contractile force (Fenn 1940). Later on, the ionic mechanisms underlying the resting membrane potential (resting E_M_) and action potential (AP) were elucidated, which allowed further development of the concept of a central role for K^+^ in muscle fatigue. In brief, the K^+^ loss from muscle fibers occurs over several APs due to the K^+^ efflux associated with the AP repolarization phase, resulting in an increased extracellular [K^+^] ([K^+^]_e_) and lowered intracellular [K^+^] ([K^+^]_i_). As the [K^+^] gradient across the sarcolemma decreases, resting E_M_ depolarizes (for details see next section entitled “Sarcolemmal resting E_M_ and action potentials”). As a consequence of the depolarization, voltage sensitive Na^+^ channels (Na_V_ channels) become inactivated, reducing AP amplitude and slowing AP propagation along the sarcolemma and into t-tubules. For this review, any physiological conditions leading to either a reduction in AP amplitude or a complete loss in the capacity of the sarcolemma to generate an AP compared to AP measured in unfatigued and normal physiological conditions is considered a decrease in membrane excitability. As membrane excitability is reduced due to the K^+^-induced depolarization, less Ca^2+^ is released by sarcoplasmic reticulum (SR) resulting in reduced muscular force or work, i.e. fatigue.

Over the years, this concept gained tremendous support and K^+^ shifts are still considered an important factor in the mechanism of muscle fatigue. However, studies have demonstrated that: (1) by itself, the lower intra-to-extracellular K^+^ gradient, and hence depolarization, may in many cases not be sufficient to explain the contraction-induced decrease in membrane excitability and force; (2) moderate increases in [K^+^]_e_ actually potentiates sub-tetanic force; and (3) changes in Cl^−^ conductance (G_Cl_) may be crucial in determining when K^+^ can either potentiate or depress force. A first objective is to review how perturbations in K^+^, Na^+^ and Cl^−^ gradients across the muscle membrane during muscle activity affect membrane excitability and force and to discuss whether the changes in gradients are sufficient to contribute to the decrease in membrane excitability and force as originally conceptualized. A second objective is to discuss the long-ignored phenomenon of K^+^-induced force potentiation and consider its physiological role at the onset of moderate exercise. A third objective is to discuss the link between metabolism and membrane excitability. The fourth and final objective is to propose an evolved concept integrating the dual roles of K^+^ (i.e., potentiation and depression) during muscle activity and fatigue and how they are modulated or controlled by Na^+^, Cl^−^ ClC-1 channels, K_ATP_ channels and Na^+^,K^+^-ATPase (Na^+^,K^+^-pump, NKA).

## Sarcolemmal resting E_M_ and action potentials

### Resting E_M_

E_M_ is a diffusion potential that is created when ions cross the sarcolemma through their channels. Thus, the sarcolemma must be permeable to the ions, which must have a concentration gradient across the sarcolemma to contribute to E_M_. In resting skeletal muscle, [K^+^]_i_, measured in different species and muscles, is approximately 160 mM (ranging between 90 and 190 mM), whilst the interstitial [K^+^] ([K^+^]_int_), measured mostly in human muscles, is typically just over 4 mM (Table 1, also see review by (McKenna et al. 2023). This large K^+^ gradient favors muscle K^+^ efflux making E_M_ more negative. Early studies using amphibian muscles demonstrated that resting E_M_ mostly behaves like a K^+^ electrode (Hodgkin and Horowicz 1959; Adrian 1956). This conclusion was based on the fact that the steady state resting Em became depolarized when [K^+^]_e_ is increased and hyperpolarized with a decreased [K^+^]_e_; the new steady state resting E_M_ values being close to the expected values calculated from the Nernst equation. However, close examination of the data reveals that the resting E_M_-[K^+^]_e_ slope in these studies was not completely identical to the expected Nernst potential (Table 2). For amphibian muscles, the calculated slope is 40 mV per decade change in [K^+^]_e_ instead of 59 mV as predicted by the Nernst equation in the Adrian (1956) study. In the Hodgkin and Horowicz (1959) study, the slope was 51 mV. Notably, the differences in the measured and expected slope are even greater for mammalian muscles (Table 2). At 25 °C and 37 °C, the expected slopes are 59 and 62 mV, respectively, whereas the measured slopes ranged from 28 to 47 mV. This deviation of the slope from expected values involves effects from Na^+^, Cl^−^ and electrogenic contribution of NKA.

**Table 1:** Changes in interstitial and intracellular K^+^ concentrations at rest and after fatigue

Species	Muscle	Rest (R)	Fatigue (F)	[K^+^]_F_ [K^+^]_R_	References
[K^+^]_int_ (mM)					
Human	Gastrocnemius	4.1	7.5	1.8	(Green et al. 1999)
Human	Gastrocnemius	4.4	11.8	2.7	(Green et al. 2000)
Human	Vastus lateralis	4.2	9.0	2.1	(Juel et al. 2000)
Human	Vastus lateralis	4.4	10.3	2.3	(Nielsen et al. 2004a)
Human	Vastus lateralis	4.4	13.7	3.1	(Street et al. 2005)
Human	Vastus lateralis	4.1	11.4	2.8	(Mohr et al. 2004)
Human	Vastus lateralis	4.2	11.9	2.8	(Nordsborg et al. 2003)
Human	Vastus lateralis^**1**^	3.9	5.5	1.4	(Lott et al. 2001)
Human	Vastus lateralis	4.0	10.5	2.6	(Gunnarsson et al. 2013)
Cat	Triceps surae^**2**^	4.0	4.8	1.2	(MacLean et al. 1998)
Mouse	Gastrocnemius	5.2	8.8	1.7	(Radzyukevich et al. 2009)
[K^+^]_i_ (mM)					
Frog	Semitendinosus	142	97	0.68	(Balog and Fitts 1999)
Human	Vastus lateralis	161	141	0.88	(Sjøgaard 1983)
Human	Vastus lateralis	162	129	0.80	(Sjøgaard et al. 1985)
Human	Vastus lateralis	125	110	0.88	(Gunnarsson et al. 2013)
Mouse	EDL soleus	182 168	134 136	0.74 0.81	(Juel 1986)
Mouse	Soleus	174	145	0.83	(Juel 1988)
Rat	EDL soleus	150 131	85 96	0.57 0.73	(Nagaoka et al. 1994)
Rat^**3**^	Soleus plantaris white gastrocnemius red gastrocnemius	90 109 125 114	64 93 86 87	0.71 0.85 0.70 0.76	(Lindinger and Heigenhauser 1987)
Rat^**4**^	Soleus plantaris white gastrocnemius red gastrocnemius	118 141 143 147	112 122 130 128	0.95 0.87 0.91 0.87	(Lindinger et al. 1987)

Concentration values are the reported mean using the unit as per each study. [K^+^]_int_ and [K^+^]_i_ were reported after a fatigue bout, except for two studies: ^**1**^Exercise at 60% VO_2max_. ^**2**^Twitch contractions at 5 Hz. For [K^+^]_i_ measurements, fatigue was elicited in vitro by field stimulation with two exceptions; ^**3**^muscles were stimulated in situ with intact blood flow and ^**4**^[K^+^]_i_ was measured following swimming

**Table 2:** [K^+^]-resting E_M_ relationship in unfatigued skeletal muscles

Species	Muscle	Temperature	[K^+^] (mM)	ΔResting E_M_/Δdecade [K^+^] (mV)	References
From change in [K^+^]_e_					
Frog	Sartorius	13–23 °C	0.5–5.0	40^C^	(Adrian 1956)
Frog	Semitendinosus	20 °C	5.0–50	51^C^	(Hodgkin and Horowicz 1959)
			0.5–10^**1**^ 10–100^**1**^	34^C^ 55^C^	
Frog	Sartorius	25 °C	3–12	59	(Renaud and Light 1992)
Mouse	EDL soleus	25 °C	4–14 4–14	51^C^ 48^C^	(Cairns et al. 1997)
Mouse	EDL soleus	25 °C	4–11	45^C^ 48^C^	(Cairns and Borrani 2015)
Mouse	Red sternomastoid	37 °C	3–100	28	(Dulhunty 1977)
Mouse	EDL soleus	37 °C	4–11 4–11	37 45	(Yensen et al. 2002)
Mouse	EDL soleus diaphragm	37 °C	4–15 4–15 4–15	46 47 44	(Ammar et al. 2015)
Mouse	Soleus	37 °C	4–10	24	(Uwera et al. 2020)
Rat	Soleus	30 °C	4–14	39^C^	(Cairns et al. 1995)
From change in [K^+^]_i_					
Frog	Sartorius	13–23 °C	110–190	50	(Adrian 1956)

Resting E_M_ was determined after manipulating either [K^+^]_e_ or [K^+^]_i_ over the range of [K^+^] as indicated in the table. The change in resting E_M_ per decade change in [K^+^] are as reported or calculated (C) using the data from individual resting E_M_ and [K^+^] values. ^**1**^Relationship obtained in Cl^−^ free solutions

In the resting state, the sarcolemmal permeability to Na^+^ is very small, which was estimated to be 1% of the K^+^ permeability (Hodgkin and Horowicz 1959). This Na^+^ permeability is due to a very small proportion of Na_V_ channels being open at rest; a fact that is confirmed in some studies by small hyperpolarization in the presence of tetrodotoxin (TTX) or in Na^+^ free saline solutions (Yensen et al. 2002; Nastuk and Hodgkin 1950; Huxley and Stampfli 1951) but not all (Cairns et al. 2003; Overgaard et al. 1997). As demonstrated by Hodgkin and Horowicz (1959), Na^+^ causes only small resting E_M_ depolarization when [K^+^]_e_ ranged between 2 and 10 mM.

Cl^−^ is the third ion of importance affecting resting E_M_. Contrary to K^+^ and Na^+^, the Cl^−^ distribution across the sarcolemma is in equilibrium with the resting E_M_ in amphibian muscles; i.e., the Cl^−^ equilibrium potential (E_Cl_) and resting E_M_ are the same (Hodgkin and Horowicz 1959). As a consequence, there is no net Cl^−^ flux at rest. For mammalian muscles, some studies also reported an equilibrium for Cl^−^ with resting E_M_ similar to amphibian muscles, while others reported an E_Cl_ that was less negative than resting E_M_ (Aickin et al. 1989; Donaldson and Leader 1984; Dulhunty 1978; Geukes Foppen 2004). As recently reviewed (Pedersen et al. 2016), studies reporting an E_Cl_ less negative than resting E_M_ may have used experimental conditions, such as hypertonic extracellular solutions, that increase the activity of some active Cl^−^ transport, such as the secondary active Na^+^, K^+^, Cl^−^ co-transporter, allowing an accumulation of intracellular Cl^−^. As a consequence of this Cl^−^ accumulation, a net Cl^−^ efflux occurs, which results in small Cl^−^-induced depolarization. In another study, intracellular [Cl^−^] ([Cl^−^]_i_) was measured under several conditions involving changes in [Cl^−^]_e_ and [K^+^]_e_ either with or without a constant [K^+^]_e_ [Cl^−^]_e_ product; in all cases the changes in [Cl^−^]_i_ were as predicted for passive Cl^−^ distribution across the sarcolemma (McCaig and Leader 1984). Thus, it is likely that in mammalian muscles E_Cl_ and resting E_M_ are the same.

Contrary to the K^+^ effect, changes in [Cl^−^]_e_ only cause transient changes in resting E_M_ (Hodgkin and Horowicz 1959; Cairns et al. 2004). For example, lowering [Cl^−^]_e_ from 120 to 30 mM caused a depolarization from −99 to −78 mV as Cl^−^ left the sarcoplasm, followed by a hyperpolarization back to −99 mV as [Cl^−^]_i_ decreased (Hodgkin and Horowicz 1959). A major Cl^−^ effect is the stabilization of resting E_M_ from two points of view. First, it reduces the extent and rate of depolarization when [K^+^]_e_ is increased. For example, exposing red sternomastoid fibers to 60 mM K^+^ resulted in a 25 mV depolarization within 3 min in the presence of Cl^−^ compared to a 32 mV depolarization within 1 min when Cl^−^ was replaced by sulfate (Dulhunty 1978). Second, in the absence of Cl^−^, muscle fibers become myotonic; i.e., they spontaneously generate AP in the absence of any stimulation (Bretag 1987; Lehmann-Horn and Jurkat-Rott 1999).

Thus, while changes in [Cl^−^]_e_ only causes transient change in resting E_M_, the Cl^−^ effect on reducing the K^+^-induced depolarization is another factor that reduces the [K^+^]_e_-E_M_ slope from the expected Nernst potential.

Most of the measured [K^+^]_e_-E_M_ slopes in Table 2 are less than the expected slope calculated from the Nernst potential. The mean difference (± standard error) between measured and expected slopes is 10.2 ± 4.7 mV for amphibian muscles (13–25 °C), which is not that different from 11.8 ± 2.0 mV for mammalian muscles (25 °C). However, the mean difference is higher in mammalian muscles at 37 °C, being 22.3 ± 3.5 mV. This greater difference may partially be related to a greater contribution of the electrogenic NKA as it transports three Na^+^ out of and two K^+^ into the sarcoplasm as well as a greater activity at higher temperatures. Indeed, the NKA electrogenic contribution to resting E_M_ increases from 10 mV at 19 °C (Hicks and McComas 1989) to 15–20 mV at 37 °C (Ammar et al. 2015; Chibalin et al. 2012).

Overall, resting E_M_ depends primarily on the distribution of three ions and the activity of one active transport. The sarcolemma behave as a K^+^ electrode, for which any changes in the [K^+^] gradient result in a new steady state resting E_M_. Changes in [Cl^−^] gradient, on the other hand, causes transient change in resting E_M_ while Na^+^ has a very small effect as the sarcolemma is almost impermeable to this ion at rest. NKA also contributes to resting E_M_ because of (i) its electrogenic nature and (ii) the maintenance of the [Na^+^] and [K^+^] gradients.

### Action potentials

APs in muscle fibers are triggered when sarcolemmal E_M_ reaches a threshold, defined as the E_M_ at which Na_V_ channels start to open resulting in a subsequent increase in Na^+^ conductance (G_Na_) allowing large Na^+^ influx that rapidly depolarizes the sarcolemma from −70 mV to + 30 mV. The repolarization back to −70 mV depends on (i) Na_V_ channel fast inactivation to stop the depolarization and (ii) the activation of voltage-sensitive K^+^ channels (K_V_) that increases G_K_ and K^+^ efflux, allowing E_M_ to return to its original resting level (Hodgkin and Huxley 1952).

Although it is well established that AP kinetics depend primarily on Na_V_ and K_V_ channel characteristics, a Cl^−^ effect should not be ignored. The role of Cl^−^ in membrane excitability has been extensively reviewed (Pedersen et al. 2016), so here the role of Cl^−^ on sarcolemmal and t-tubular excitability is briefly discussed. Skeletal muscle expresses the Cl^−^ ClC-1 channel, a member of the large ClC family of Cl^−^ channels (Jentsch et al. 2002). ClC-1 channels are active at rest providing a G_Cl_ that is 2 to 4-times greater than the K^+^ conductance (G_K_) in amphibian muscles and 5 to 9-times greater than G_K_ in mammalian muscles (Pedersen et al. 2009b; Hodgkin and Horowicz 1959; Sperelakis 1969; Dulhunty 1979). The fraction of open ClC-1 channels is high at rest and remains nearly constant during a single AP. This is because AP only lasts 1–2 ms while the depolarization-induced activation of ClC-1 channels has time constants of 40 and 450 ms (Weiss and Magleby 1990; Fahlke and Rüdel 1995).

ClC-1 channels allow for Cl^−^ influx during both depolarization and repolarization phases of the AP because as soon as E_M_ depolarizes it becomes less negative than E_Cl_, which favors a net Cl^−^ influx (Pedersen et al. 2016). This is supported by at least three studies. First, current–voltage relationship using rat psoas muscles show an outward Cl^−^ current upon membrane depolarization, which indicate a Cl^−^ influx (Fahlke and Rüdel 1995). Second, removing Cl^−^ from the bathing solutions or exposing mechanically rat EDL skinned fibers to 9-anthracene carboxylic acid (9-AC), a ClC-1 channel blocker, result in more negative AP threshold; this suggests that the Cl^−^ influx provide an outward current counteracting any depolarizing stimulus to trigger an AP, at least in t-tubules (Dutka et al. 2008). Third, removing Cl^−^ or the presence of 9-AC also prolong the AP repolarization phase in the t-tubules of frog semitendinosus muscle and in rat EDL t-tubules providing evidence for Cl^−^ influx that can contribute to the repolarization phase with K^+^ (Heiny et al. 1990; Dutka et al. 2008). However, under normal conditions with normal resting [K^+^]_e_, reducing [Cl^−^]_e_ from 120 to 10 mM has little impact on AP kinetics (Cairns et al. 2004), possibly because the increase in G_Na_ is such that the Na^+^ inward current overwhelmingly exceeds the Cl^−^ outward current that opposes the Na^+^-induced depolarization. However, this is not the case under conditions of high [K^+^]_e_ (see section on “*Modulation of the K*^+^*-induced force depression by changes in G*_*Cl*_”).

A major consequence of the Na^+^, K^+^ and Cl^−^ fluxes during APs are changes in both intra- and extracellular Na^+^, K^+^ and Cl^−^ concentrations, which then affect resting E_M_, membrane excitability and consequently force. To fully understand these processes, we must first look at the magnitude of these ionic changes during muscle activity and fatigue.

## Ionic disturbances during muscle contractions and fatigue

Initially, plasma ion concentrations were measured in the venous blood derived from contracting muscles. The advent of the microdialysis technique, however, has enabled the determination of the interstitial ion concentrations. Notably, during fatiguing exercise increases in [K^+^]_int_ are 4–7 mM greater than increases in venous plasma [K^+^] (Green et al. 2000; Juel et al. 2000; Nielsen et al. 2004a; Street et al. 2005). The changes in plasma [K^+^], skeletal muscle [K^+^]_int_ and [K^+^]_i_ with exercise have recently been extensively reviewed (McKenna et al. 2023). So here, we briefly summarize changes in interstitial and intracellular ion concentrations, being closest to the sarcolemma and being the ion concentrations affecting resting E_M_ and AP.

Muscle [K^+^]_int_ have been primarily measured in human studies, albeit there are two studies with animal muscles. In resting muscles, [K^+^]_int_ varies between 4.1 and 4.4 mM, increasing to peak value of 7.5 to 13.7 mM during or immediately after fatiguing muscle contractions, while less intense muscle contractions resulted in smaller increases (Table 1). Muscle [K^+^]_i_ values have, in contrast, mostly been reported in studies of isolated muscles from rats, mice or frogs and vary largely among studies; e.g., resting values range from 90 to 182 mM. Although the extent of the decrease in [K^+^]_i_ with fatigue was quite variable, most studies (~ 80% in Table 1) reported decreases in [K^+^]_i_ ranging from 1.1- to 1.5-fold. In line with this, in two human studies, vastus lateralis muscle [K^+^]_i_ decreased from 161 to 129 mM in one study and to 141 mM in the other following exhaustive exercise (Sjøgaard 1983; Sjøgaard et al. 1985).

While both plasma and [K^+^]_int_ are increased substantially during muscle contractions (Table 2, McKenna et al. 2023), the situation is different for Na^+^. For example, one study in exercising humans reported a 15 mM decrease in [Na^+^]_int_, whilst venous [Na^+^] increased by 8 mM (Street et al. 2005). The increase in venous [Na^+^] occurs because of greater water than Na^+^ flux from plasma into muscle interstitial fluid (Sjøgaard et al. 1985; Lindinger et al. 1994), whilst the decrease in [Na^+^]_int_ is because of this interstitial fluid influx, together with lower water than Na^+^ shift from muscle interstitium to fiber intracellular space. At rest, [Na^+^]_i_ ranged between 10 and 29 mM and increased with fatigue in most studies, with values ranging from no change to a threefold increase (Table 3). Although there is a Cl^−^ influx during APs, the few reports of activity-induced changes in [Cl^−^]_i_ in mammalian muscles are inconsistent (Table 4).

**Table 3:** Changes in interstitial and intracellular Na^+^ concentrations at rest and after fatigue

Species	Muscle	Rest (R)	Fatigue (F)	[Na^+^]_F_ [Na^+^]_R_	References
[Na^+^]_int_ (mM)					
Human	Vastus lateralis	143	128	-0.1	(Street et al. 2005)
[Na^+^]_i_ (mM)					
Frog	Semitendinosus	16	49	3.1	(Balog and Fitts 1996)
Human	Vastus lateralis	22	24	1.1	(Sjøgaard 1983)
Human	Vastus lateralis	13	23	1.8	(Sjøgaard et al. 1985)
Mouse	Soleus	13	23	1.8	(Juel 1986)
Mouse	Soleus	11	15	1.4	(Juel 1988)
Rat	EDL soleus	18 28	66 62	3.7 2.2	(Nagaoka et al. 1994)
Rat	EDL soleus tibialis	19 29 29	34 36 36	1.8 1.9 1.2	(Everts et al. 1993)
Rat^**1**^	Soleus plantaris white gastrocnemius red gastrocnemius	28 24 12 23	27 27 16 29	0.96 1.1 1.3 1.3	(Lindinger and Heigenhauser 1987)
Rat^**2**^	Soleus plantaris white gastrocnemius red gastrocnemius	26 11 14 10	33 14 16 13	1.3 1.3 1.2 1.3	(Lindinger et al. 1987)

Concentration values are the reported mean as per each study. For [Na^+^]_i_ measurements, fatigue was elicited in vitro by field stimulation with two exception; ^**1**^muscles were stimulated in situ with intact blood flow and ^**2**^[Na^+^]_i_ was measured following swimming

**Table 4:** Changes in intracellular Cl^−^ concentration at rest and after fatigue

Species	Muscle	Rest (R)	Fatigue (F)	[Cl^−^]_F_ [Cl^−^]_R_	References
[Cl^−^]_i_ (mM)					
Human	Quadriceps femoris	21.9	25.8	1.2	(Sahlin et al. 1978)
Human	Quadriceps femoris	15.1	28.0	1.9	(Bergström et al. 1971)
Human	Quadriceps femoris	8.8	9.1	1.1	(Kowalchuk et al. 1988)
Rat^**1**^	Soleus plantaris white gastrocnemius red gastrocnemius	24 17 11 15	23 26 11 23	0.96 1.5 1.0 1.5	(Lindinger and Heigenhauser 1987)
Rat^**2**^	Soleus plantaris white gastrocnemius red gastrocnemius	11 7 9 6.4	13 9 11 9.5	1.2 1.3 1.2 1.5	(Lindinger et al. 1987)
Rat^**1**^	Soleus plantaris white gastrocnemius red gastrocnemius	13.5 8.6 7.7 9.7	22.3 23.1 12.0 13.1	1.7 2.7 1.6 1.4	(Lindinger and Heigenhauser 1988)

Concentration values are the reported mean as per each study. Fatigue was elicited as follows: ^**1**^perfused muscles were stimulated in situ; ^**2**^[Cl^−^]_i_ was measured following swimming

## Contribution of the ionic disturbances to changes in resting E_M_** and action potential**

### ***Resting E***_***m***_

E_M_ measurements in muscle fibers are complicated by muscle movements during contraction often causing microelectrode damage or dislodgment out of the fiber. Very few studies continuously recorded E_M_ during the fatigue bout; most studies measured E_M_ after fatigue. Among the latter, several E_M_ measurements were carried out at various times during the recovery allowing an extrapolation of E_M_ back to the last contraction of the fatigue bout. However, for studies in which data is only provided for the period after fatigue, one must take into account that some recovery may have resulted in some underestimation of the E_M_ changes during fatigue.

Some studies reported no sarcolemmal depolarization in human intercostal, rat intercostal and extensor carpi radialis longus muscles, when continuously stimulated at 10 Hz for 30 min (Hanson 1974a, b) (Table 5, bottom section). However, for both studies, twitch force either did not decrease (human muscle) or increased (rat muscle); so based on this, it is unlikely that there was any large extent of fatigue. In another study (Hicks and McComas 1989), for which changes in force was not reported, a 12 mV hyperpolarization was observed in rat soleus muscle stimulated with 4 s long train of 20 Hz pulses every 5 s for 5 min. When NKA activity was reduced with either an exposure to the specific inhibitor ouabain or with a reduction in temperature from 37 °C to 19 °C, resting E_M_ then depolarized by 4–5 mV during muscle activity. The authors concluded that the hyperpolarization during muscle activity under control conditions was because of an increased NKA activity contributing to resting E_M_.

**Table 5:** Resting E_M_ and AP overshoot at rest and post stimulation

Species	Muscle	Electrical stimulation	Change in force	°C	Resting E_M_ (mV)		Overshoot (mV)		References
					Rest	Post	Rest	Post	
Substantial fatigue									
Frog	Lumbrical^1^	C: 70 Hz, 30 s	−89%	22 °C	−85	−50	20		(Lannergren and Westerblad 1986)
Frog	Lumbrical I^1^ II III	C: 70 Hz, 30 s	−60% −60% −60%	22 °C	−90 −89 −85	−75 −65 −70			(Westerblad and Lannergren 1986)
Frog	Lumbrical^1^ Detubulated	C: 70 Hz, 30 s	Not measured	22 °C	−79 −82	−46 −71	32 36	−11 11	(Lannergren and Westerblad 1987)
Frog	Sartorius^2^	I: 80 Hz, 1TPS, 3 min	−82%	22 °C	−82	−68	32	30	(Renaud and Mainwood 1985a)
Frog	Sartorius^2^	I: 80 Hz, 1TPS, 3 min	See Note #4	22 °C	−87	−68			(Renaud and Mainwood 1985b)
Frog	Sartorius^2^	I: 80 Hz, 1TPS, 3 min	−81%	22 °C	−85	−71			(Renaud 1989)
Frog	Semitendinosus^2^	I: 150 Hz, 1TPS, 5 min	−90%	22 °C	−84	−77	19	10	(Balog et al. 1994)
Frog	Semitendinosus^2^	I: 150 Hz, 1TPS, 5 min	See Note #5	22 °C	−83	−74	20	7	(Balog and Fitts 1996)
Frog	Sartorius^3^	I: 200 Hz, 1TPS, 3 min	−89%	22 °C	−86	−78	26	24	(Light et al. 1994)
Mouse	EDL^3^ Soleus^3^	I: 40 Hz, 1TPS, 1 min	−90% −71%	37 °C	−75 −70	−56 −58			(Juel 1986)
Mouse	Soleus^3^	I: 140 Hz, 1TPS, 3 min	−70%	37 °C	−78	−83			(Matar et al. 2000)
Mouse	FDB^1^	I: 140 Hz, 1TPS, 3 min	See Note #6	37 °C	−82	−58			(Cifelli et al. 2008)
Mouse	Soleus^2^	I: 40 Hz, 0.3 TPS, 5 min	−60%	35 °C	−70	−57			(Juel 1988)
Rat	Plantaris^2^	I:50 Hz, 0.4 TPS, 60 min	−71%	37 °C	−79	−71			(Karelis et al. 2005)
No fatigue									
Frog	EDL^3^	C: 10 Hz, 60 s	180%	20–24 °C			17	28	(Hanson and Persson 1971)
Human	Intercostal^3^	C: 10 Hz, 30 s	0%	37 °C	−80	−81	24	22	(Hanson 1974a)
Rat	Intercostal^3^	C: 10 Hz, 30 s	174%^C^	37 °C	−77	−75	30	24	(Hanson 1974a)
Rat	Extensor carpi radialis longus^3^	C: 10 Hz, 30 s	157%^C^	37 °C	−77	−73	26	25	(Hanson 1974b)
Rat	Soleus^2^	I:20 Hz, 0.8 TPS, 5 min	Not measured	37 °C 19 °C	−80 −80	−92 −80	3	6 18	(Hicks and McComas 1989)

The table top part reports changes in resting E_M_ and overshoot following substantial fatigue; i.e., when the decrease in force was at least 60%. The bottom portion reports changes in resting E_M_ and overshoot when there was either no or increase in twitch force. Muscles were either ‘C’ continuously stimulated at the indicated frequency and duration or ‘I’ intermittently stimulated to trigger tetanic contractions with the indicated frequency during the train in Hz, the number of train per sec (TPS) and fatigue period in min. ^**1**^E_M_ measurements were continuously carried out during the fatigue bout. ^**2**^E_M_ measurements were carried out before and at various times after the fatigue bout allowing an extrapolation of E_M_ data to the time of the last contraction. ^**3**^E_M_ measurements were carried out before and after the fatigue bout. ^**4**^Tetanic forces were reported in the accompanying paper (Renaud and Mainwood 1985a) ^**5**^Force was not measured in the study but authors referred to an earlier study by Balog et al. (1994). ^**6**^ Tetanic [Ca^2+^]_i_ but not force was measured

In contrast to the abovementioned studies, large sarcolemmal depolarization in both amphibian and mammalian muscles has been observed when there were large decreases in force; i.e., evidence of fatigue (Table 5, top section). The mean depolarization from all these studies is 16 mV, with a range from 7 to 35 mV. Changes in [K^+^] gradient across the sarcolemma is most likely the major component responsible for the depolarization. However, since most studies did not concomitantly measure changes in resting E_M_, [K^+^]_int_ and [K^+^]_i_, here we estimate the extent of the K^+^ contribution to the depolarization in mammalian muscles by using mean values (± standard error) from Table 2. Mean [K^+^]_int_, from human studies, and [K^+^]_i_, from mostly animal studies, are in resting muscles respectively 4.3 ± 0.1 and 134.1 ± 6.8 mM, giving rise to a Nernst potential for K^+^ (E_K_) of -92 mV. After fatigue, the values are respectively 10.1 ± 0.9 mM, 108.1 ± 5.9 mM and -63 mV. This implies that mean E_K_ decreases by 29 mV; mostly from the increase in [K^+^]_int_ (~ 23 mV, 79%) compared to the decrease in [K^+^]_i_ (~ 6 mV, 21%). More importantly, the 29 mV decrease in E_K_ is almost twice as large as the 16 mV resting E_M_ depolarization. This is further supported by one study in which resting E_M_, [K^+^]_int_ and [K^+^]_i_ were concomitantly measured reporting a 24 mV decrease in E_K_ versus a 12 mV resting E_M_ depolarization (Juel 1986). Thus, changes in [K^+^] gradient contribute to the sarcolemmal depolarization, but the extent of the depolarization is considerably less than that of E_K_. This is most likely because of the NKA electrogenic contribution (Juel 1986; Hicks and McComas 1989) and the Cl^−^ stabilizing effect on resting E_M_ that reduces the K^+^-induced depolarization as discussed above under resting conditions.

### Action potentials

The above studies for which there was no force loss and depolarization with muscle activity (Table 5 bottom), also reported no change in AP overshoot (the term overshoot was originally used to reflect the fact that during an AP the E_M_ peak became positive; i.e., the overshoot normally represents the peak E_M_ above 0 mV. For this review the term overshoot will refer to the AP E_M_ peak value even when under some conditions it remains negative). Studies describing large resting E_M_ depolarization and force decrease also reported decreases in AP overshoot, from as small as 2 mV to as large as 43 mV (Table 5, top). Two mechanisms detailed below are involved in the decrease in AP overshoot: (i) the decrease in [Na^+^] gradient and (ii) a depolarization-induced Na_V_ channel inactivation.

A decrease in [Na^+^] gradient reduces the Na^+^ current during the depolarization phase leading to smaller AP overshoot. For most experiments presented in Table 3 (15 out of 19 measures), the [Na^+^] gradient decreased by 1.1 to 1.8-fold and for a minority of studies (4 out of 19 measures) the decrease exceeded twofold. Increasing [Na^+^]_i_ (Desmedt 1953) or decreasing [Na^+^]_e_ (Nastuk and Hodgkin 1950) in amphibian muscles to mimic changes in [Na^+^] gradient, resulted in lower AP overshoot. In mouse soleus muscle, 1.5 and 2.5-fold decreases in [Na^+^]_e_ had little effect on resting E_M_ while it reduced AP overshoot by 10 and 20 mV, respectively (Cairns et al. 2003). These studies then support the notion that decreases in [Na^+^] gradient can contribute to a decrease in AP overshoot.

Depolarization-induced Na_V_ channel inactivation, fast and slow, is a second mechanism for overshoot depression during fatigue. Fast inactivation occurring in ms is crucial for (i) stopping the depolarization phase so that the subsequent K^+^ efflux can repolarize the membrane and (ii) allowing the unidirectional AP propagation. Studies on steady state fast inactivation reported that 10% of Na_V_ channels are fast inactivated when resting E_M_ is on average −90 mV (range between studies from −105 to −70 mV); a value increasing to 50% at −67 mV (range from −87 to −50 mV) and with all Na_V_ channels fast inactivated at −40 mV (range from −60 to −20 mV) (Cummins and Sigworth 1996; Cummins et al. 1993; Bendahhou et al. 2002, 1995, 1999; Hayward et al. 1996, 1997, 1999; Webb et al. 2009; Wu et al. 2014; Kuzmenkin et al. 2003; Rojas et al. 1999). Slow inactivation occurs over seconds to minutes and is believed to be an important regulator of Na_V_ channel activity during muscle activity. Studies on steady state slow inactivation, in which Na_V_1.4 channel, the Na_V_ channel expressed in skeletal muscle, is transfected in HEK-293 cells and oocytes, reported that 10% of Na_V_1.4 channels were slow inactivated at −90 mV, being 50% at an average of−63 mV (range between studies from −70 to −60 mV) and with all Na_V_1.4 channels being slow inactivated at −10 mV (from −20 mV to 0 mV). Other studies using skeletal muscle fibers reported more hyperpolarized steady state slow inactivation; i.e., 10% decrease at −107 mV (range from −120 to −90 mV), 50% decrease at −90 mV (range from −110 to −70 mV) and no current at −60 mV (range −80 to −50 mV) (Featherstone et al. 1996; Ruff 1996, 1999; Ruff et al. 1988; Kirsch and Anderson 1986; Simoncini and Stuhmer 1987). Ruff (Ruff et al. 1988) has proposed that slow inactivation of Na^+^ channels is important in the decreased sarcolemmal excitability with fatigue. If depolarization-induced slow as well as fast inactivation plays a role in the decrease in AP amplitude, then the E_M_ range for which AP amplitude decreases should correspond to the E_M_ range for which fast and slow inactivation occurs.

One approach to induce membrane depolarization is to increase [K^+^]_e_. For mouse soleus and extensor digitorum longus (EDL) muscles, AP amplitude decreased by 50% at resting E_M_ of −70 and −60 mV, respectively (Ammar et al. 2015; Wang et al. 2022). Maximum rate of depolarization, often used as an index of Na^+^ current (Hodgkin and Katz 1949), decreased by 50% at −64 mV (Uwera et al. 2020). Thus, the E_M_ range at which AP amplitude and Na^+^ current is reduced by 50% correspond to the E_M_ range at which fast inactivation reaches 50% as mentioned above. It also corresponds to the E_M_ range for 50% slow inactivation when Na_V_1.4 channels are expressed in HEK-293 cells and oocytes. Similarly, muscle fibers do not generate AP when resting E_M_ becomes less negative than −50 mV (Ammar et al. 2015; Wang et al. 2022; Uwera et al. 2020) corresponding to the E_M_ range at which there is complete Na_V_1.4 channel fast inactivation. Taken together, these studies supports the notion that decreases in AP amplitude/overshoot during fatigue are in part due to the sarcolemmal depolarization triggering Na^+^ channel inactivation.

**Fig. 1:**
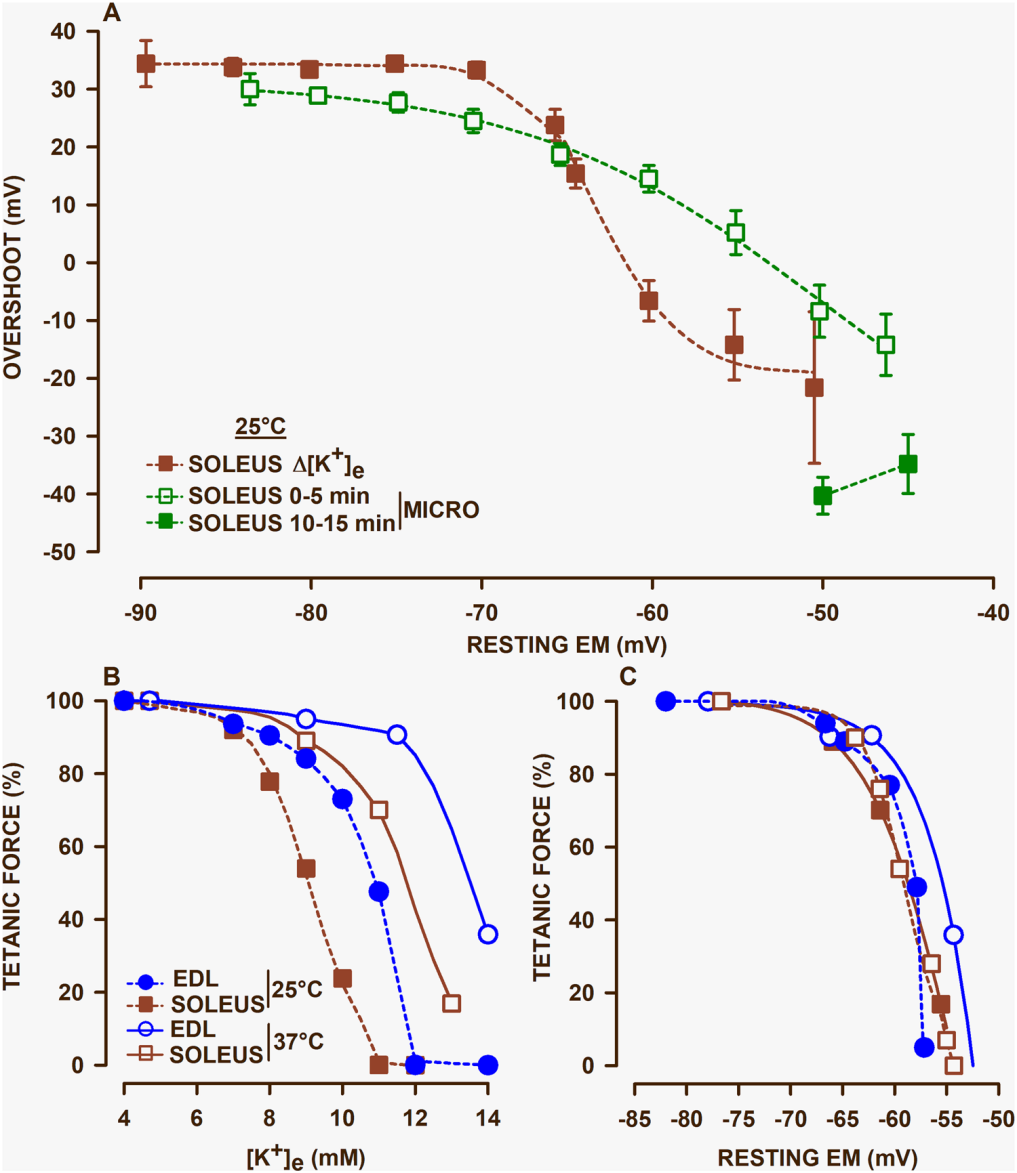
**A** Action potential overshoot-resting E_M_ relationship, **B** mean tetanic force-[K^+^]_e_ relationship and **C** mean tetanic force-mean resting E_M_ relationship in mouse EDL and soleus muscles. **A** Resting E_M_ were measured at different (Δ) [K^+^]_e_ after tetanic force had reached a steady state (over a 30–40 min period) or at 4 mM K^+^ but in fibers for which resting E_M_ depolarized over a 5 min period before remaining stable over another 10 min period following microelectrode (micro) penetration. Resting E_M_ were separated in bin of 5 mV and overshoot were averaged (vertical bars being standard error; not visible if smaller than symbols); data from (Cairns et al. 2022). **B** and **C** Data at 25 °C from Cairns et al. 1997; data at 37 °C from Ammar et al. 2015

One issue with the K^+^-induced depolarization approach is the long time required for tetanic force to reach a new steady state following an increase in [K^+^]_e_, i.e. up to 30–40 min. Usually AP are captured in fibers for which resting E_M_ remains stable upon microelectrode penetration. However, in a recent study (Cairns et al. 2022), APs were also measured in fibers exposed to 4 mM [K^+^]_e_ that did not maintain a constant resting E_M_ upon microelectrode penetration. The depolarization lasted ~ 5 min before staying constant over the next 10 min, the latter suggesting that the initial five min depolarization was due to some shifts in [K^+^] and [Cl^−^] gradients as opposed to microelectrode-induced sarcolemmal damages that would have caused much greater depolarization. Figure 1A compares the overshoot-resting E_M_ relationship measured during the short depolarization period (i.e., 0 to 5 min microelectrode-induced depolarization) and the prolonged depolarization (K^+^-induced depolarization). Mean overshoots were quite similar between the two measurements for resting E_M_ from −90 to −67 mV. At resting E_M_ less negative than −67 mV, overshoot depression was greater with the K^+^-induced than the microelectrode-induced depolarization. Despite a stable resting E_M_ 10–15 min after the microelectrode penetration, overshoot continued to decrease, reaching values that were even less negative than those measured at elevated [K^+^]_e_. For example, at resting E_M_ of −50 mV, mean AP overshoot was −8 mV after 5 min of depolarization further decreasing to −40 mV for the 10–15 min period during which resting E_M_ remained at −50 mV. The most likely mechanism responsible for the further decrease in overshoot while resting E_M_ remained is slow inactivation that can take up to 20 min to reach steady state (Webb et al. 2009). Cairns et al. (2022) also reported another important observation; that is a complete lack of inexcitable fibers at all resting E_M_ during the microelectrode-induced depolarization compared to several fibers becoming inexcitable for the K^+^-induced depolarization. Considering that most fatigue protocols involve stimulation periods of 5 min or less, the Cairns et al. (2022) study raises the possibility that the resting E_M_ at which large decreases in AP amplitude, and thus force, most likely occur at more depolarized resting E_M_ than what we have so far estimated from K^+^-induced depolarization as shown in Fig. 1C. Nevertheless, all these studies still support fast/slow inactivation as a second mechanism responsible for the decrease in AP overshoot during fatigue.

In summary, resting E_M_ depolarizes when there is evidence of fatigue; i.e., there is a decrease in force and this depolarization is primarily the result of a reduced [K^+^] gradient. As a consequence of the depolarization, the extent of Na_V_ channel inactivation increases, resulting in lower AP overshoot/amplitude, which is further lowered by a reduced [Na^+^] gradient. There is now abundant evidence for a decrease in SR Ca^2+^ release and reduced active [Ca^2+^] ([Ca^2+^]_i_), defined as the [Ca^2+^]_i_ during contraction, as a major mechanism by which force decreases during fatigue (Allen et al. 1989, 2008a; Lee et al. 1991; Westerblad and Allen 1991). The question is whether the K^+^ and Na^+^ changes associated with muscle APs are large enough to be a major cause for the reduced Ca^2+^ release and force during fatigue.

## The K^+^-induced force depression

Decreases in active [Ca^2+^]_i_ have been demonstrated in frog semitendinosus and mouse FDB muscle fibers with concomitant decrease in shortening capacity in mouse FDB when [K^+^]_e_ is increased (Lucas et al. 2014; Quinonez et al. 2010). More importantly, a recent study has clearly demonstrated a relationship between AP overshoot and active [Ca^2+^]_i_ in which active [Ca^2+^]_i_ remains stable from + 30 to 0 mV, decreasing rapidly as AP peak became less than 0 mV, with no Ca^2+^ release by −40 mV (Wang et al. 2022). The changes in and effects of K^+^ on resting E_M_ and AP and the relationship between AP and Ca^2+^ release strongly support a role for K^+^ in the decrease in force during fatigue. However, the K^+^-force relationships for twitch and tetanic contractions are more complicated than originally thought. For tetanic contractions, there is a range of [K^+^]_e_ for which tetanic force remains close to maximal (≥ 90%) despite significant resting E_M_ depolarization and lower overshoot, until a [K^+^]_e_ is reached above which force declines abruptly. For twitch and sub-maximal tetanic contractions, small increases in [K^+^]_e_ actually potentiate force, while at higher [K^+^]_e_, twitch and sub-maximal tetanic force are decreased. In this section, we discuss the K^+^-induced force depression on tetanic force and how it is modulated by changes in [Na^+^] gradient, G_Cl_ and NKA activity, then followed by a subsequent section discussing K^+^-induced potentiation.

### ***Tetanic force-[K***^+^***]***_***e***_*** relationship***

The maximum force a muscle can generate is measured during a completely fused tetanus. For frog sartorius and mouse EDL muscles at 25 °C, peak tetanic force decreases by less than 10–15% when [K^+^]_e_ is raised from 4 to 9 mM and is completely abolished at 12 mM [K^+^] (Fig. 1B) (Renaud and Light 1992; Cairns et al. 1997). The critical [K^+^]_e_, defined as the [K^+^]_e_ above which tetanic force drops abruptly, is 9 mM for these two muscles. Notably, mouse soleus is more sensitive to raised [K^+^]_e_ as the critical [K^+^]_e_ is only 7 mM and no force is generated at 11 mM (Fig. 1B). The differences between mouse soleus and EDL tetanic force-[K^+^]_e_ relationships persist at 37 °C, but with higher critical [K^+^]_e_, being 9 and 12 mM for soleus and EDL, respectively (Fig. 1B) (Ammar et al. 2015). Similarly for rat muscles at 30 °C, the critical [K^+^]_e_ for soleus and EDL were 9 and 11 mM, respectively (Hansen et al. 2005; Pedersen et al. 2003; Cairns et al. 1995). Soleus is known as a slow-twitch fatigue resistant muscle primarily composed of type 1 fibers, being 87% of all fibers in rat and 67% in mouse; the remaining fibers being type IIA fibers (Banas et al. 2011; Armstrong and Phelps 1984). EDL, on the other hand, is a fast twitch fatigable muscle with fibers being composed primarily of type IIB (56–57% in rat and mouse) and IIX (46% in mouse). Therefore, the most fatigue resistant type I and IIA fibers have lower critical [K^+^]_e_ values than the fatigable type IIB and IIX fibers.

Another observation from the above studies was that the higher the experimental temperature the higher the critical [K^+^]_e_. This was especially confirmed in rat soleus as the critical [K^+^]_e_ values were 8, 9 and 10 mM at 20°, 30° and 35 °C, respectively (Pedersen et al. 2003). There is one study reporting faster and greater K^+^-induced force depression at 37 °C than at 25 °C when mouse soleus were exposed to 10 mM [K^+^]_e_ (Cairns et al. 2011). It is more than likely, however, that the critical [K^+^]_e_ increases with temperature from two points of view. First, the K^+^-induced depolarization is less at 37 °C than at 25 °C (Table 2). For mouse EDL, the depolarization per decade change in [K^+^]_e_ is 48 mV at 25 °C and 42 mV at 37 °C; for soleus, the values are respectively 45 and 37 mV. Thus, one should expect that less depolarization at 37 °C results in a higher critical [K^+^]_e_. Second, as discussed above, the NKA electrogenic contribution is greater at 37 °C, being 15–20 mV, than at 19 °C, being 10 mV (Hicks and McComas 1989; Ammar et al. 2015; Juel 1986; Chibalin et al. 2012). More importantly, if NKA activation with salbutamol reduces the rate and extent of the force loss at high [K^+^]_e_ and increases the critical [K^+^]_e_ (Andersen and Clausen 1993; Clausen et al. 1993; Clausen and Everts 1991; Pedersen et al. 2003), then greater NKA activity at 37 °C should give rise to slower and smaller force loss at 37 °C than at 25 °C for a given increase in [K^+^]_e_.

The most important aspect to consider is the tetanic force-resting E_M_ relationship because the K^+^ effect is not direct but via a depolarization of the sarcolemma. Muscle [K^+^]_int_ have been primarily measured in human studies. For frog sartorius, mouse EDL and soleus muscles at 25 °C, tetanic force remains constant from -95 to −70 mV, drops by about 10% from −70 to −65 mV and reaches zero between −60 and −55 mV (Fig. 1C) (Renaud and Light 1992; Cairns et al. 1997, 2022); i.e., 90% of the force loss occurs over a 5–10 mV range. Notably, the differences between EDL and soleus for the tetanic force-[K^+^]_e_ relationship shown in Fig. 1B are no longer observed for the tetanic force-resting E_M_ relationship as shown in Fig. 1C; i.e., the greater K^+^ sensitivity of soleus is due to greater depolarization at a given [K^+^]_e_. The tetanic force-resting E_M_ relationship of mouse soleus is basically the same at 25 °C and 37 °C, while it is slightly shifted toward less negative resting E_M_ for EDL. The small difference in tetanic force-resting E_M_ relationship between EDL and soleus at 37 °C may be related to smaller decreases in overshoot when resting E_M_ becomes less negative than −70 mV (Ammar et al. 2015).

It is important to note that the tetanic force-resting E_M_ relationships in Fig. 1C are derived from studies using K^+^-induced depolarization. If we now take into consideration the Cairns et al. 2022 study discussed above, the critical resting E_M_ at which force decreases abruptly may be more depolarized than what is shown in Fig. 1C. That is, from the AP overshoot-resting E_M_ relationship obtained with K^+^-induced depolarization a sudden drop in AP peak to −10 mV occurred when resting E_M_ dropped from −65 to −60 mV (Fig. 1A). Under the same conditions, the critical resting E_M_ for the abrupt tetanic force decrease occurred between −60 mV and −55 mV (Fig. 1C) suggesting that it occurs when AP peak is −10 mV or more negative. For the shorter microelectrode-induced depolarization, a decrease in AP peak to −10 mV occurred at −50 mV (Fig. 1A) representing a 10 mV shift toward less negative resting E_M_ compared to the K^+^-induced depolarization. Assuming that tetanic force abruptly declines once AP peak becomes −10 mV or more negative, then for shorter depolarization period the critical resting E_M_ for abrupt tetanic force loss would be −50 mV instead of −60 mV. Thus, to fully understand the role of K^+^ in fatigue, future studies are necessary to determine the full time course of the AP depression following increases in [K^+^]_e_ as opposed to when tetanic force reaches a steady state after more than 30 min.

The next question is whether the changes in [K^+^]_e_ and [K^+^]_i_ during fatigue by itself can be considered a major factor in the mechanism of fatigue. In comparing the relationships between [K^+^]_e_, resting E_M_, AP and force in frog sartorius, Light et al. (1992) demonstrated that while an increase in [K^+^]_e_ to 7 mM in unfatigued sartorius muscles mimicked changes in resting E_M_ and AP during fatigue, the increased [K^+^]_e_ had little effect on tetanic force of unfatigued muscle. Furthermore, increasing [K^+^]_e_ to 7.5 mM immediately after a fatigue bout did not reduce recovery of force after fatigue despite preventing a recovery of resting E_M_ (Comtois et al. 1994). In humans, during 30 min moderate (30 Watts, W) knee extension exercise, mean [K^+^]_int_ increased to 10 mM within 5 min but then decreased to a steady state level ranging between 7 and 9 mM; mean [K^+^]_int_ reached 9.7 mM during exhaustive exercise (Nielsen et al. 2004a). Thus, the difference in peak [K^+^]_int_ between a non- exhausting and exhausting exercise was not only small, but slightly less in exhaustive exercise. Most studies in which [K^+^]_int_ was measured by microdialysis in human reported a mean value not exceeding 12 mM (Table 1). Although more studies are needed for [K^+^]_int_ in animal muscles, changes during fatigue in [K^+^]_i_ of human vastus lateralis muscles are within the range reported for mouse and rat muscles (Table 1). So, if the changes during fatigue in [K^+^]_int_ (measured in human muscles) and if the critical [K^+^]_e_ (measured in mouse and rat muscles) are representative of the situation in human, mouse and rat muscles, then 12 mM [K^+^]_int_ at 37 °C is expected to reduce tetanic force by 50% in fatigue resistant muscles such as the soleus, but having little effect in fatigable muscles such as the EDL. Human muscle has a mixed fiber type composition, so the expected effects would be intermediate between these two different types of muscles. Overall, these results do not strongly support the concept that perturbations in K^+^ (i.e., both [K^+^]_int_ and [K^+^]_i_) per se are not a major mechanism for the decrease in force during fatigue at least in muscles with low fatigue resistance. Further studies are necessary to determine [K^+^]_int_ in animal models as well as the critical [K^+^]_e_ in human muscles. However, as discussed below, one cannot exclude K^+^ as a potential factor in muscle fatigue without looking at an interaction with Na^+^, Cl^−^ and NKA.

### ***Modulation of the K***^+^***-induced force depression by Na***^+^

Although studies reported faster and greater extent of force decrease when amphibian and mammalian muscles were fatigued at lowered extracellular [Na^+^] ([Na^+^]_e_) compared to control conditions (Cairns et al. 2003; Bezanilla et al. 1972), one must also determine if the [Na^+^]_e_ and [Na^+^]_i_ changes during fatigue significantly affect force in unfatigued muscles. In frog sartorius, mimicking a reduction in [Na^+^] gradient observed with fatigue (Table 3) by lowering [Na^+^]_e_ by 1.2 and twofold (i.e., from 120 to 100 and 60 mM) reduced peak force by 10% and 30%, respectively (Bouclin et al. 1995). Mammalian muscles are more resistant to a decrease in [Na^+^]_e_. In rat soleus and mouse EDL and soleus, a twofold reduction in [Na^+^]_e_ from 147 to 75 mM had no effect on tetanic force as significant decreases in tetanic force occurred at [Na^+^]_e_ below 40 mM, i.e., a 3.8-fold decrease in [Na^+^] gradient (Overgaard et al. 1997, 1999; Cairns et al. 2003). Considering that most studies report a less than twofold decrease in [Na^+^] gradient during fatigue (Table 3), it would appear that the change in [Na^+^] gradient is by itself in most cases too small to be of any major importance in the force decrease during fatigue, despite its effects on AP as discussed above.

Concomitant changes in Na^+^ and K^+^ gradients, on the other hand, have a synergistic depressive effect on tetanic force; i.e., their combined effects are greater than their additive effects. In frog sartorius, tetanic force decreased by about 8% when either [K^+^]_e_ was increased from 3 to 7 mM to mimic a 2.3-fold decrease in [K^+^] gradient, or when [Na^+^]_e_ was decreased from 120 to 110 mM to mimic a 1.2-fold decrease in [Na^+^] gradient. If the Na^+^ and K^+^ effects were additive, the concomitant change in Na^+^ and K^+^ gradient should lower force by 15% whereas a much greater 31% decrease was actually observed (Bouclin et al. 1995). In rat soleus muscle, tetanic force decreased by 10% when [K^+^]_e_ was increased from 4 to 9 mM (2.3-fold reduction in the [K^+^] gradient) and remained constant when [Na^+^]_e_ was reduced from 147 to 85 mM (1.7-fold in the [Na^+^] gradient); concomitant changes of both gradients resulted in a 50% force reduction (Overgaard et al. 1999). Finally, in mouse soleus, a 2.0-fold increase in [K^+^]_e_ from 4 to 8 mM decreased tetanic force by 9% and a 1.5-fold decrease in [Na^+^]_e_ (from 147 to 100 mM) reduced force by 3%,while concomitant changes in [K^+^]_e_ and [Na^+^]_e_ resulted in a force depression of 40%, more than threefold greater than a calculated additive effect of 12% (Cairns et al. 2022). Noticeably, Cairns et al (2022) reported that a similar concomitant change in [K^+^] and [Na^+^] had an additive and not a synergistic depressive effect on single AP, albeit the effect may be different for a train of APs. Furthermore, they reported that 15% of soleus fibers became inexcitable when [K^+^]_e_ was increased from 4 to 8 mM while a decrease in [Na^+^]_e_ from 147 to 100 mM had no effect; concomitant changes in both [Na^+^]_e_ and [K^+^]_e_ resulted in 20% of fibers becoming inexcitable suggesting that Na^+^ and K^+^ have a small synergistic effect on sarcolemmal excitability. Thus, reductions in either [Na^+^] or [K^+^] gradients observed during fatigue have by themselves limited adverse impact on tetanic force, whereas concomitant reductions in [K^+^] and [Na^+^] gradients result in tetanic force decreases that are large enough to suggest that the combined changes in [K^+^] and [Na^+^] gradient are important in the mechanism of muscle fatigue.

### ***Modulation of the K***^+^***induced force depression by changes in G***_***Cl***_***.***

As discussed in the section on AP, there is a net Cl^−^ influx during both AP depolarization and repolarization phases (Dutka et al. 2008; Heiny et al. 1990; Fahlke and Rüdel 1995). Under normal resting [K^+^]_e_ of ~ 4 mM and [Na^+^]_e_ of ~ 147 mM, reducing [Cl^−^]_e_ to 10 mM had no long-lasting effect on resting E_M_, AP or tetanic force in unfatigued mouse soleus, while it increased the rate of fatigue (Cairns et al. 2004). As discussed in the section on resting E_M_, a major effect of Cl^−^ is a slower and lower extent of the K^+^-induced membrane depolarization; this effect implies that a decrease in [Cl^−^]_e_ or of G_Cl_ should increase the K^+^-induced force depression and thus the rate of fatigue. However, a series of studies demonstrated that the Cl^−^ effects are more complex.

In one study, an increase of [K^+^] to 11 mM at a normal pH_e_ of 7.4 reduced tetanic force and M-wave (an extracellular measurement of APs from the muscle surface) to 20–25% of the initial values measured at 4 mM (Pedersen et al. 2005). The extracellular pH (pH_e_) was then lowered from 7.4 to 6.8 by raising CO_2_ in the gas phase from 5 to 24%, in order to reduce G_Cl_, as Cl^−^ ClC-1 channels are pH-sensitive (Hutter and Warner 1967; Palade and Barchi 1977). Following the decrease in pH_e_ to 6.8, both tetanic force and M-wave area increased to 80–90% of initial values at 4 mM [K^+^]_e_ and pH_e_ 7.4. Likewise, at 9 mM [K^+^]_e_, the same decrease in pH_e_ increased the number of excitable fibers from 48 to 94% and AP overshoot by 10 mV (Pedersen et al. 2005). Accordingly, lowering pH_e_ from 7.4 to 6.8 shifted the tetanic force-[K^+^]_e_ relationship by 2 mM toward higher [K^+^]_e_. These acidic pH_e_ effects were associated with a 46% reduction in G_Cl_ (with no effect on G_K_). Mimicking the reduction in G_Cl_ by lowering [Cl^−^]_e_ as well as by exposing soleus to 9-AC at pH_e_ 7.4 had the same effect on force and M-wave as the low pH_e_. The authors concluded that a partial decrease in G_Cl_ is the mechanism by which acidic pH_e_ caused an increase in tetanic force and M-wave area during the K^+^ -induced depolarization.

The above conclusion was further supported by another study in which mechanically skinned fibers with intact t-tubules were exposed to various [K^+^]_i_ in order to alter t-tubular resting E_M_ (Pedersen et al. 2004). When contractions were elicited with electrical stimulations to trigger APs in t-tubules, a decrease in intracellular pH (pH_i_) from 7.1 to 6.6 shifted the force-[K^+^]_i_ relationship toward lower [K^+^]_i_, i.e., more depolarized t-tubules. A similar shift was not observed when i) Cl^−^ was removed from the bathing solution and ii) when contractions were elicited via an activation of the voltage sensor (also known as Ca_V_1.1 channel or dihydropyridine receptor). The authors concluded that greater t-tubular depolarization was necessary to induce force loss in the presence of Cl^−^ (or G_Cl_) in acidic than in normal pH_i_.

In a third study (de Paoli et al. 2013), rat soleus muscles were stimulated for 30 s train at 60 Hz. Under those conditions, force reached a plateau in about 2 s and decreased constantly thereafter. The extent of the depolarization between APs became greater as [Cl^−^]_e_ was decreased stepwise from 127 to 0 mM. Despite greater depolarization, the rate at which force decreased became slower when [Cl^−^]_e_ was lowered from 127 to 60 mM (to lower G_Cl_) and then became faster from 60 to 0 mM Cl^−^. The authors concluded that any decrease in G_Cl_ worsens the K^+^-induced depolarization whereas small decrease in G_Cl_ improves membrane excitability and tetanic force while large decrease in G_Cl_ worsens the K^+^-induced decrease in membrane excitability and force by mechanisms explain below.

The mechanism of action by which decreases in G_Cl_ affects membrane excitability and tetanic force as [K^+^]_e_ increases have been extensively reviewed (Nielsen et al. 2017; Pedersen et al. 2016). Briefly, three issues must be taken into account. First, when a stimulation, either electrical during an experiment or at the neuromuscular junction following acetylcholine binding to its receptor, depolarizes the membrane toward AP threshold, there is a constant Cl^−^ influx that counteracts the stimulation-induced depolarization. AP threshold becomes less negative following prolonged depolarization, induced either by higher [K^+^]_e_ or continuous stimulations as Na_V_ channels become inactivated. As a consequence of a less negative threshold, greater stimulation current is needed to reach it. Lowering G_Cl_ reduces the Cl^−^ influx that opposes the stimulatory depolarization. This explains why the number of excitable fibers increases at 9 mM K^+^ when G_Cl_ is lowered by decreasing pH_e_. Second, as discussed in the section on AP, there is a constant Cl^−^ influx during AP depolarization and repolarization phases. Under normal conditions, G_Na_ during the AP depolarization is substantial and largely overwhelms the counteracting Cl^−^ current that opposes the depolarization; i.e., the G_Na_:G_Cl_ ratio is very high. This is no longer the case when Na_V_ channels are inactivated by prolonged membrane depolarization. However, small decreases in G_Cl_ has two opposing effects: it allows (i) for greater K^+^-induced depolarization and (ii) greater G_Na_:G_Cl_ ratio. If small decrease in G_Cl_ improves AP amplitude and force at raised [K^+^]_e_, then one can suggest that the increase in AP amplitude due to greater G_Na_:G_Cl_ ratio largely overcomes the expected lower AP amplitude due to the greater K^+^-induced depolarization. Third, there is an optimum decrease in G_Cl_ for which the extent of the depressive effects of any depolarizations on excitability and force is at its lowest. As shown by de Paoli et al. (2013), small decreases in G_Cl_, induced by decreases in [Cl^−^]_e_ from 127 to 60 mM, reduce the extent of the depressive effects of any membrane depolarization because the increased G_Na_:G_Cl_ ratio improves AP threshold and allows greater AP depolarization. Further decreases in G_Cl_ not only worsen the K^+^-induced depolarization but it may do it to the point at which the depolarization depressive effects as Na_V_ channel inactivation becomes too great resulting in further decrease in membrane excitability and force.

### ***Modulation of the K***^+^***-induced force depression by NKA***

NKA is largely responsible for the maintenance of the [Na^+^] and [K^+^] gradients across the muscle membrane. The NKA mechanisms of action, molecular isoforms and activity regulation in muscle have been extensively reviewed (Pirkmajer and Chibalin 2016; Clausen 2003, 2013; McKenna et al. 2023). Here, we briefly discuss how NKA, its activation and inhibition, modulates the K^+^ effects on force depression. Exposing unfatigued soleus muscles to 12.5 mM K^+^ reduced tetanic force to zero within 20 min, while in the presence of 10 µM ouabain, a NKA-specific inhibitor, the decrease occurred in only 2 min; conversely, NKA activation with 10 µM salbutamol, a β_2_-adrenergic receptor agonist, reduced the rate of force decrease, reaching zero after 40 min (Clausen and Everts 1991). Slower force decrease also occurred when NKA was activated by insulin, epinephrine and calcitonin gene related peptide (CGRP) (Andersen and Clausen 1993; Clausen and Everts 1991; Clausen et al. 1993; Clausen and Flatman 1977). Furthermore, activating NKA after force had decreased to a steady level at elevated [K^+^]_e_ or after a concomitant increase in [Na^+^]_i_ and decrease in [K^+^]_i_ allowed for large force recovery (Andersen and Clausen 1993; Clausen et al. 1993; Macdonald et al. 2005; Pedersen et al. 2003). Improvement of tetanic force in the presence of salbutamol correlated with improvement of M-waves, which suggest an improvement of membrane excitability (Overgaard et al. 1999). Thus, activation of NKA has the capacity to reduce the rate and extent of the K^+^-induced force depression in unfatigued skeletal muscle.

NKA activity increases during muscle activity (see review by (McKenna et al. 2023). This for example was shown as 2 Hz stimulation for 10 min or 60–120 Hz stimulation for 10 s increased ouabain-suppressible Na^+^ efflux and K^+^ influx in rat soleus muscle (Everts and Clausen 1994; Nielsen and Clausen 1997). More importantly, stimulating soleus muscle with 1.5–2 s long 30 Hz tetanic contractions every min after force had been depressed at 10 mM K^+^ allowed for full force recovery; for soleus exposed to 12.5 mM K^+^ the stimulation allowed for a partial recovery (Overgaard and Nielsen 2001; Nielsen et al. 1998). The force recovery was associated with a partial recovery of resting E_M_ and membrane excitability, with the latter determined by M-waves. Furthermore, Nielsen et al. (1998) provided evidence that resting E_M_ and force recovery were associated with increases in NKA activity brought about by the release of CGRP from neurons innervating skeletal muscle. Salbutamol, epinephrine, insulin and CGRP, all NKA activators, substantially reduced the rate at which force declined when rat soleus was continuously stimulated at 60 Hz for min while exposed at various [K^+^]_e_ (Clausen and Nielsen 2007). Finally, when soleus muscles were stimulated with 400 ms long tetanic stimulation at 40 Hz every 3rd s for 5 min and compared to control, 10 µM, terbutaline, a β_2_-adrenergic receptor agonist, reduced the extent of the resting E_M_ depolarization by 35%, the [K^+^]_i_ decrease by 31%, the [Na^+^]_i_ increase by 25% and the force decrease by 10% (Juel 1988). Juel (1988) suggested that the terbutaline effects involved a NKA activation. Thus, muscle contractions induce NKA activation, which then has the capacity to minimize perturbations in muscle E_M_, [K^+^]_i_, [Na^+^]_i_ and force.

In resting unfatigued skeletal muscle, the electrogenic NKA contribution to resting E_M_ under normal [K^+^]_e_ conditions (i.e., 4–5 mM K^+^) is 12–20 mV in EDL, soleus and diaphragm muscles (Ammar et al. 2015; Chibalin et al. 2012; Clausen and Flatman 1977). Furthermore, several studies have reported that under normal [K^+^]_e_ conditions and in the resting state, a 3 to 9 mV hyperpolarization occurs when NKA is activated by β_2_-adrenergic receptor agonists or insulin (Clausen and Flatman 1977; Kuba 1970; Kuba et al. 1978; Kuba and Nohmi 1987; van Mil et al. 1995; Juel 1988). Finally as discussed above, muscle contractions increase NKA activity, which then modulates resting E_M_ and membrane excitability (Juel 1988; Hicks and McComas 1989; Nielsen et al. 1998; Overgaard and Nielsen 2001). Thus, one mechanism of action for NKA is via its electrogenic effects making resting E_M_ more negative and counteracting the K^+^-induced depolarization and the subsequent decrease in force.

It is important to note, however, that while NKA activation during muscle activity with or without an exposure to catecholamines leads to more negative resting E_M_ and smaller force loss, the same does not always apply when resting muscles are exposed to elevated [K^+^]_e_. First, some studies reported that in rat diaphragm, mouse soleus and lumbrical muscles the extent of the catecholamine-induced hyperpolarization decreased as [K^+^]_e_ was increased; the hyperpolarization near 0 mM [K^+^]_e_ being 10–20 mV and becoming zero at 10 mM [K^+^]_e_ (Uwera et al. 2020; Kuba and Nohmi 1987; van Mil et al. 1995). In the study of Uwera et al. (2020), salbutamol triggered an increase in tetanic force at 10 mM [K^+^]_e_ in soleus muscle despite no effect on resting E_M_. Second, an exposure of resting muscle under normal [K^+^]_e_ conditions (4–5 mM) to catecholamines/agonists results in a hyperpolarization (Clausen and Flatman 1977; Kuba 1970; Kuba et al. 1978; Kuba and Nohmi 1987; van Mil et al. 1995; Juel 1988) and increase in twitch force (Holmberg and Waldeck 1980; Reading et al. 2003; Cairns et al. 1995, 1993; Bowman and Zaimis 1958; Cairns and Dulhunty 1993a, b). However, the hyperpolarization cannot be the mechanism by which twitch force increases because the changes in resting E_M_ are not within the range that affects twitch force; i.e., under normal conditions resting E_M_ is more negative than −75 mV while twitch force depression occurs when resting E_M_ becomes less negative than −60 mV. Together these results suggest that the mechanism of action by which catecholamine improves force, regardless of [K^+^]_e_, cannot be solely due to an effect on resting E_M_. Indeed, catecholamines also increases Ca^2+^ release. This mechanism involves (i) phosphorylation of SR Ca^2+^ release channels, known as the ryanodine receptors (RyR1) and (ii) in some muscles, such as diaphragm and amphibian muscle but not mammalian limb muscles, a phosphorylation of the t-tubular voltage sensor/Ca^2+^ Ca_V_1.1 channels (for more details see review by Cairns and Borrani 2015).

Overall, NKA activation by muscle contraction, catecholamines and CGRP is most likely crucial at protecting skeletal muscle from the K^+^-induced force depression. As discussed in greater detail in the section below entitled “*A new perspective about the role of K*^+^*, Na*^+^ *and Cl*^*−*^* on muscle performance from the onset of exercise to fatigue*”, this protection is important at the onset of, or during mild exercise, when [K^+^]_int_ is high but there is no metabolic stress triggering fatigue.

## The K^+^-induced force potentiation

The observation that increased [K^+^]_e_ can potentiate twitch force of skeletal muscle was made as early as 1935, where Anna Baetjer reported an increase in twitch force in cat muscle upon arterial infusion of K^+^-enriched Ringer, an effect that was attributed to an increase in muscle rather than nerve function as it was present also in curarized muscle (Baetjer 1935). In a subsequent paper, which described the phenomenon of post-tetanic potentiation, Brown and Euler performed experiments in which arterial KCl infusion of cat tibialis anterior muscle induced either increased or decreased twitch force depending on the K^+^ dose delivered (Brown and von Euler 1938). Thus, the basic observation of a biphasic concentration dependent effect of K^+^ on twitch force was present in the literature already more than 80 years ago. Since then, sporadic reports of K^+^-induced force potentiation have appeared in the literature (Walker 1948; Holmberg and Waldeck 1980; Cairns et al. 1997, 2011; Renaud and Light 1992; Pedersen et al. 2019; Olesen et al. 2021; Yensen et al. 2002; Overgaard et al. 2022; Lannergren and Westerblad 1986) where the phenomenon has been observed in many vertebrate animal species, including cats, mice, rats, frogs, guinea pigs and also humans (Grob et al. 1957). The magnitude of K^+^ potentiation depends on [K^+^]_e_ and is observed between 6 and 14 mM in various preparations (Pedersen et al. 2019; Olesen et al. 2021; Lannergren and Westerblad 1986; Yensen et al. 2002). The fiber type of the preparation may be an important determinant of the magnitude of force potentiation and the degree of [K^+^]_e_-elevation needed to evoke the response as shown by Pedersen et al. 2019, where [K^+^]_e_ elevation from 4 to 8 mM provided a maximal response of 17% twitch potentiation in slow twitch dominant rat soleus muscles, while an elevation to 11 mM was needed for full twitch potentiation (60% increase) in fast twitch dominant rat EDL muscles. Similarly, the K^+^-induced twitch potentiation was more pronounced in EDL than in soleus muscles of guinea pigs (Holmberg and Waldeck 1980). However, in mice, EDL and soleus muscles showed approximately the same capacity for twitch potentiation (Yensen et al. 2002). In all three species, the EDL muscles reached maximal twitch potentiation and exhibited potentiation at higher [K^+^]_e_ compared to soleus, indicating a fiber type effect on the dose–response relationship between twitch force and [K^+^]_e_.

Another notable feature of the K^+^-potentiation phenomenon is the reliance on stimulation frequency. As such, K^+^-induced potentiation is most prominent in twitch contractions evoked by a single stimulus, where relative improvements of up to 100% have been observed. Further, [K^+^]_e_-induced potentiation has also been observed to increase maximal force of partially fused tetanic contractions evoked by low frequency stimulation (Pedersen et al. 2019; Olesen et al. 2021; Holmberg and Waldeck 1980). In contrast, the [K^+^]_e_ that lead to K^+^-induced twitch potentiation invariably do not potentiate maximal force of fully fused tetanic contractions evoked at high frequencies; K^+^-induced twitch potentiation may even occur concomitantly with small depression of tetanic force (Pedersen et al. 2019; Overgaard et al. 2022; Olesen et al. 2021). Interestingly, however, recent reports suggest that rate of force development in high-frequency tetanic contractions may be enhanced by moderate elevations of [K^+^]_e_ (e.g., to 7–10 mM) (Overgaard et al. 2022; Olesen et al. 2021) and [K^+^] increase may also enhance peak force of doublet-induced contractions with a short interspike interval corresponding to up to 300 Hz in rat muscle (Olesen et al. 2021) but not in mouse muscle (Overgaard et al. 2022). Furthermore, moderate [K^+^]_e_-elevations have been shown to potentiate power in dynamic contractions of rat muscle (Pedersen et al. 2019). Taken together, moderate [K^+^]_e_ elevation broadly enhances several important aspects of muscle contractile function and may therefore be considered as a potentially important positive modulator of muscle contractile function during exercise.

The possible mechanisms behind K^+^-induced potentiation have been investigated in only a few studies. In 2002, Yensen et al. considered the possibility that the increased force was a consequence of a broadened AP seen during elevation of [K^+^]_e_. However, the authors dismissed this possibility since an experimentally induced non-K^+^ related broadening of the AP did not lead to potentiation of twitch-responses in mouse muscle. In accordance, Wang et al. (2022) found that increasing [K^+^]_e_ to 16 mM induced an initial increase in Ca^2+^ release and twitch force followed by a decrease in both variables; the increase in Ca^2+^ release occurs as the sarcolemmal depolarized from −80 to −65 mV while the subsequent decrease started once resting E_M_ fell to less than −65 mV. However, changes in the time integral of the AP was not related to the initial increases in force and Ca^2+^, but did correlate strongly with the subsequent reduction in force and Ca^2+^ release.

In a recent report, Overgaard et al. (2022) re-addressed an old notion originally proposed more than 80 years ago by Brown & Euler (1938) that K^+^-potentiation shared mechanistic pathways with post-tetanic potentiation. More recent knowledge links post-tetanic potentiation to phosphorylation of the regulatory light chain of myosin, but since mouse muscles devoid of the enzyme that phosphorylates myosin light chain could still produce K^+^-induced potentiation of a normal magnitude and since K^+^-induced potentiation and post-tetanic potentiation were additive, it was concluded that the two potentiation phenomena were mechanistically distinct (Overgaard et al. 2022). The most convincing clues to the mechanism behind K^+^-induced potentiation comes from studies that link the contractile potentiation to an increase in intracellular Ca^2+^ transients, which again is consequent to membrane-depolarization (Quinonez et al. 2010; Pedersen et al. 2019). The proposal that K^+^ induced potentiation is related to an increase in Ca^2+^ transients fits well with the frequency dependence of K^+^-potentiation, where potentiation is observed in contractions evoked by a single stimulus (twitch) or by a low-frequency train, both of which fall on the steep portion of the Ca^2+^-tension relationship. In contrast, there is no potentiation during fully fused contractions, which lie on the plateau of the Ca^2+^-tension relationship.

The link between depolarization at rest and increased Ca^2+^ transients during activation, is however, still not elucidated. Possibly the small increase in resting [Ca^2+^]_i_ that is observed following [K^+^]_e_ elevation could be involved in the pathway (Quinonez et al. 2010), since higher resting [Ca^2+^]_i_ would enhance the Ca^2+^ binding of intracellular Ca^2+^ buffers and pave the way for a faster rise in free active [Ca^2+^]_i_ when SR Ca^2+^ release channels are activated. However, such a link has yet to be proven (Pedersen et al. 2019).

In studies demonstrating K^+^-potentiation the effect typically develops within 2–10 min of placing a muscle in increased [K^+^]_e_ (Pedersen et al. 2019; Overgaard et al. 2022). It seems likely this time-course corresponds to the development of depolarization, which depends on the size of the muscle preparation and, hence, the diffusion distance to the center of the preparation (Cairns et al. 1995). However Yensen et al., (2002) observed a more gradual development of potentiation over 90 min with small increases in [K^+^]_e_ and other studies have shown that the time-course for the decrease in force following large increases in [K^+^]_e_ may require up to 30–90 min to reach a new steady state. A major reason for such slow development is that depolarization is delayed by the membrane potential-clamping effect of a large G_Cl_ (de Paoli et al. 2013; Dulhunty 1978). A functional implication of this could be that during moderate intensity exercise, when [K^+^]_int_ is accumulated gradually, potentiation may be the dominant effect of elevated K^+^ early in an exercise session, while K^+^-induced force depression will occur later because it requires further K^+^ accumulation and full development of the associated depolarization to elicit reduction in excitability.

### Modulators of K^+^-induced potentiation

It is of interest to determine whether various “exercise factors” could modulate the magnitude of K^+^-induced potentiation as was the case for the K^+^-induced force depression. Temperature is one such factor. For mouse muscles, twitch potentiation reached a maximum of 20% at 25 °C (Cairns et al. 1997) while an increase of almost 100% was observed at 37 °C (Yensen et al. 2002). Although twitch force is smaller at 37 °C than at 25 °C so that relative change appears greater at 37 °C, it can be concluded that at physiologically relevant temperatures for mammalian muscles, K^+^-induced twitch potentiation is quite substantial (Olesen et al. 2021; Yensen et al. 2002).

Another exercise factor, lactic acid, reduces the Cl^−^ conductance but did not alter the degree of K^+^-induced potentiation in rat muscle (Olesen et al. 2021). Furthermore, NKA activation via β_2_-agonists, gave rise to further enhancement of twitch force in EDL muscles already potentiated by high [K^+^]_e_, but had no effect on soleus twitch force (Olesen et al. 2021).

The Na^+^ gradient may decrease during exercise, but so far, K^+^-induced potentiation has not been studied in conjunction with a reduced Na^+^ gradient. Interestingly, however, lowering the Na^+^ gradient by itself was observed to give rise to a small increase in twitch force in rat muscles (Overgaard et al. 1999), but not in mouse muscle (Cairns et al. 2003). Taken together, these abovementioned results demonstrate that exercise factors heat, lactic acid and adrenaline do not attenuate the K^+^-induced potentiation, although these factors are all known to affect the K^+^-induced force depression.

## Muscle metabolic links to sarcolemmal excitability

So far, we discussed how changes in ion gradients across the muscle membrane during fatigue affects its excitability. As discussed in the section entitled “*A new perspective for the role of K*^+^*, Na*^+^ *and Cl*^*−*^* on muscle performance from the onset of exercise to fatigue*”, fatigue is likely a protective mechanism that prevents damaging ATP depletion. Thus, it is of vital importance for the muscle to keep the balance between ATP-utilization and ATP-production in order to maintain [ATP] within a narrow range and avoid an irreversible deleterious ATP depletion (Hochachka and Matheson 1992). A link between metabolic capacity and muscle excitability may be a feed-forward signal, lowering membrane excitability and thereby restraining the muscle energy turn-over (Ørtenblad and Nielsen 2015). To support such notion we discuss three examples of how changes in metabolic status affect the activity of NKA, the ATP sensitive K^+^ channel (K_ATP_ channel) and the Cl^−^ ClC-1 channel, being an ion pump and two ion channels that affect membrane excitability.

Cells must continually regenerate ATP to keep up with demand. This is achieved by an integration of the various energy pathways and by efficient regulatory systems, ensuring that rates of ATP resynthesis are balanced with the ATP demands of exercise. Such a tight balance between ATP production and resynthesis rates is of vital importance for all cells, but particularly so in excitable cells due to their high and fluctuating energy turnover. It is remarkable how skeletal muscle fibers can instantly adjust to provide the necessary energy during exercise, where a several-fold elevated energy turnover can be sustained for hours or a more than a 100-fold increase in turn-over is obtained for short time. A number of the steps in muscle excitation–contraction-relaxation (E-C-R) coupling are either directly (ATPases) or indirectly (ion channels) dependent on muscle energy status. In contracting skeletal muscle fibers, the three main ATP consuming processes are the myosin ATPases, the SR Ca^2+^ ATPases and the Na^+^,K^+^-ATPases (NKA), which respectively consume approximately 50–60%, 40–50% and 5–10% of the energy during exercise (Clausen et al. 1991; Rolfe and Brown 1997; Ørtenblad et al. 2009). Thus, the Na^+^,K^+^-ATPase has a relatively low energy turn-over in skeletal muscle during exercise with high absolute ATPase activity.

### Exercise metabolism and ion homeostasis

Already during the early 1900’s it was established that both fat and carbohydrate can be used as fuel for metabolism during exercise and that the relative contributions of the fuels during lower exercise intensities mainly is determined by the diet (Christensen and Hansen 1939; Frentzel and Reach 1901; Krogh and Lindhard 1920; Zuntz 1896). There was also a clear knowledge of carbohydrate being the main source of substrate when exercising at high intensities, with a relationship between exercise intensity and carbohydrate metabolism. At the same time, seminal studies established the foundations of our understanding of metabolic pathways in general and herein ATP production in contracting muscle. Different glycolytic enzymes from muscle tissue were extracted and combined to artificially establish a pathway from glycogen to lactic acid (Meyerhof 1942), and with this the full glycolytic (Emben-Meyerhof) pathway, from glycogen to lactate, was elaborated by the early 1940s see (Kresge et al. 2005). Although these early studies had documented the glycolytic pathway, that carbohydrate is a major substrate during exercise and that the diet plays an important role in endurance capacity, it was not until after the introduction of the needle biopsy technique in the 1960s, that it was demonstrated that prolonged exercise performance is highly correlated with muscle glycogen contents and that exhaustion is associated with low skeletal muscle glycogen contents (Bergström et al. 1967). At present, we still have limited explanations for the mechanisms linking metabolism and muscle glycogen to muscle function. In addition to the effects of K^+^, Na^+^ and Cl^−^ discussed above, there are numerous other factors affecting muscle excitability and none has been more agreed on than metabolically derived factors affecting NKA and ion channel (i.e., K_ATP_ and ClC-1 channel) activity. It is now well established that NKA and K_ATP_ channels are primarily fueled by glycolytically-derived ATP with direct effect of muscle glycogen on muscle excitability (Dhar-Chowdhury et al. 2007).

### A causal link between metabolism and muscle excitability

The causal link between energy availability or glycogen depletion and depressed muscle function is most likely multifactorial, but studies on isolated muscles from rodents (Chin and Allen 1997) and amphibians (Stephenson et al. 1999) suggest a local factor within the muscle E-C-R coupling. The association between low muscle glycogen content and depressed contractile function was proposed that low glycogen causes a slowed glycogenolytic and glycolytic flux, compromising the required rate of ATP regeneration to sustain muscle function during exercise, referred to as the ‘energy crisis’ theory (Green 1991; Sahlin et al. 1998). Consequently, adequate ATP supply to one or more of the processes involved in E-C-R coupling cannot be maintained, leading to depressed muscle function and fatigue. The energy crisis theory is supported by observations of phosphocreatine (PCr) decreases along with increases in free ADP and IMP contents in muscles following prolonged, glycogen-depleting exercise (Norman et al. 1988; Sahlin et al. 1997).

However, the energy crisis theory is challenged by both in vitro and in vivo studies. First, a number of studies at both the cellular and whole muscle level demonstrate a strong association between low glycogen and decreased muscle function even after recovery periods, where ATP concentration would be expected to be normal (Bangsbo et al. 1992; Chin and Allen 1997). Second, muscular fatigue is also observed even when glycogen is far from depleted, or when glycogen is lowered prior to the start of exercise (Duhamel et al. 2006a, b; Ørtenblad et al. 2011). Also, intramuscular concentrations of ATP are in the range of 5–9 mM (Hargreaves and Spriet 2018; Greiner and Glonek 2021) and NKA has a high affinity for ATP with a K_1/2_ less than 0.5 mM (Blanco and Mercer 1998) and the Na^+^-stimulated NKA activity is saturated at 0.5 mM ATP (Walas and Juel 2012), suggesting that the cellular global [ATP] per se does not directly affect NKA activity (Ewart and Klip 1995). Finally, low glycogen contents also affect muscle function in the mechanically skinned fiber preparation where cellular global ATP and PCr are maintained high during contractile activity (Kabbara et al. 2000; Nielsen et al. 2009; Stephenson et al. 1999). Collectively, these series of experiments do not provide experimental support for the energy crisis hypothesis, at least at the whole cellular level.

On the other hand, one cannot exclude metabolic effects within the compartmentalised muscle fibre as [ATP] at the subcellular level inside cells may not be uniform (Jones 1986). The highly organized muscle cell forms many compartments, and hence microenvironments with high ATPase activity and restricted diffusional access of metabolites; observations on experiments utilizing whole muscles or intact fibers do not rule out a metabolic role in maintaining muscle fiber excitability. Such a functional compartmentalization of glycolytic metabolism is known in a variety of tissues, with a possible role of ATP and other metabolites regulating key steps in the muscle E-C-R coupling by delivering ATP in microenvironment of the fiber (Han et al. 1992; Korge and Campbell 1995). This would be particularly noteworthy in the muscle triad junction between the transverse tubular-system and the SR, with a diffusional restricted space around 12 nm wide, and with a high metabolic activity (Dulhunty 1984). An important conceptualization of this idea is that most of the glycolytic enzymes are connected with membranes of intracellular compartments such as the SR (Dhar-Chowdhury et al. 2007; Xu and Becker 1998) and the existence of a glycogenolytic complex associated with the SR is now well established (Entman et al. 1980; Wanson and Drochmans 1972; Xu and Becker 1998). In line with this, transmission electron microscopy imaging has revealed the existence of a heterogenic subcellular distribution of three distinct glycogen pools in skeletal muscle, indicating a requirement for distinct subcellular energy stores (Nielsen and Ørtenblad 2013). This organization places the energy stores in close vicinity to the position of utilization and provides support for the concept for highly compartmentalized energetic networks. Interestingly, it has been demonstrated that the three main energy-consuming ATPases in skeletal muscles (Na^+^,K^+^-, Ca^2+^-, and myosin- ATPases) utilize different local pools of glycogen, clearly demonstrating compartmentalized glycogen metabolism and emphasize that spatially distinct pools of glycogen are used for separated energy requiring processes (Nielsen et al. 2022). Physiologically, this ensures an effective energy transfer and regulation of energy production and utilization in the restricted cellular compartments (Nielsen and Ørtenblad 2013; Korge and Campbell 1995; Saks et al. 2008). In line with this, it has become increasingly clear that glycolytic intermediates and end-products by themselves also regulate the activity of membrane ion channels and pumps, coupling muscle energy metabolism to the regulation of ion homeostasis and cellular excitability. In this context, NKA was one of the first membrane proteins to be described to be preferentially regulated by glycolytically-derived ATP (Mercer and Dunham 1981). This concept of the fueling of the NKA by glycolytically derived ATP has later found ample support in the literature (Dhar-Chowdhury et al. 2007).

### Fueling of NKA and role in muscle excitability

There is a reasonably well established association between glycolytically-derived ATP and NKA activity and strong evidence to support the concept that glycolysis and NKA are functionally coupled. This seems to be an evolutionary conserved coupling and has been observed in several tissue types, including mammalian erythrocytes (Kennedy et al. 1986; Mercer and Dunham 1981; Schrier 1966), axons (Caldwell et al. 1960); brain synaptosomes (Erecińska and Dagani 1990), kidney cells (Lynch and Balaban 1987), smooth muscle (Campbell and Paul 1992), cardiac myocytes (Hasin and Barry 1984; MacLeod 1989; Philipson and Nishimoto 1983) and skeletal muscles (Clausen 1965; James et al. 1999a; Jensen et al. 2020; Okamoto et al. 2001). This is supported by the observation that a number of tissue types generate both pyruvate and lactate under primarily aerobic conditions in a process linking glycolytic ATP supply to NKA activity (Brooks 1986; Dhar-Chowdhury et al. 2007). This occurs in resting, well oxygenated skeletal muscles, and is closely linked to NKA stimulation by epinephrine (James et al. 1996, 1999a, 1999b; Bundgaard et al. 2003; Levy et al. 2005).

Such a tight coupling between the glycogenolytic rate and NKA activity is demonstrated by the observation that [Na^+^]_i_ decreases if glycogen breakdown is stimulated with epinephrine at rest, whilst ouabain attenuates glycogen utilization (James et al. 1999b). Also, experiments using ouabain and measurements of myoplasmic high-energy phosphates, in resting rat EDL muscles, demonstrated that NKA activity is only suppressed when glycolysis is inhibited and not affected by inhibition of oxidative phosphorylation; this suggests that normal glycolysis is the predominant source of the fuel for NKA (Okamoto et al. 2001). In support, an early study by Clausen was one of the first to demonstrate a link between muscle glycogenolysis and NKA activity, showing that glycogen utilization in resting muscle was decreased when muscle NKA activity was blocked by ouabain (Clausen 1965). Moreover, there was an apparent lactic acid production in proportion to NKA activation. It might appear that the glycolytically-derived ATP may be an inefficient metabolic pathway, as aerobic glycolysis is an inefficient means of generating ATP per unit of glucose, compared to the amount obtained by mitochondrial respiration (Vander Heiden et al. 2009; Locasale and Cantley 2011). However, the rate of glucose metabolism through aerobic glycolysis is substantially higher, with a 10–100 times faster production of lactate than the full oxidation of glucose in the mitochondria. Thus, the amount of ATP synthesized over any given period of time is comparable when either form of glucose metabolism is utilized (Shestov et al. 2014).

Also, in cell cultures, inhibition or activation of different cell membrane active pumps (such as NKA, P-glycoprotein pump, pumps involved in osmoregulation) led to reduction or increase in glycolysis, respectively, while the oxidative phosphorylation remained constant (Epstein et al. 2014). Together, available data strongly suggest that in skeletal muscle, glycolysis is the predominant source of the fuel for NKA, with a clear association of glycogenolytic/ glycolytically derived ATP on ion transport across muscle membranes. Functionally, a decreased glycogenolysis/glycolytic rate will therefore potentially adversely affect muscle excitability during contractions. Indeed, a direct link between energy state and excitability of the muscle was confirmed by blocking cross-bridge cycling and SR Ca^2+^ release with the cross-bridge cycling blockers N-benzyl-p-toluene sulphonamide and dantrolene, respectively, thereby conserving energy during repeated electrical stimulations, which in turn reduced the extent of muscle excitability loss during high-frequency stimulation (Macdonald et al. 2007).

The essential role of glycogenolytically-derived ATP on muscle excitability is substantiated in experiments using mechanically skinned fibers, enabling the maintenance of a high and constant global [ATP] during experiments under different metabolic conditions. Using this muscle fiber preparation, fatiguability induced by repeated contractions is associated with lowered glycogen contents in most experiments during both AP stimulation (Nielsen et al. 2009) and voltage sensor activation (Barnes et al. 2001; Stephenson et al. 1999), but not in all studies (Goodman et al. 2005). Further, enzymatically lowering glycogen by 70% led to a reduction in both voltage sensor activated- and AP-induced forces in skinned fibers, with larger decrease in AP-induced force by lowering glycogen (Watanabe and Wada 2019). Together, these data suggest that low glycogen and glycogenolytic rate affects the t-system polarization and excitability, as the voltage sensor inactivation is displaced to markedly more positive E_M_ values compared with AP (Na^+^ channel) inactivation (Ørtenblad and Stephenson 2003; Nielsen et al. 2004b). Taken together, studies with the mechanically skinned fiber preparation strongly demonstrate that glycogenolytically-derived energy is associated with fiber contractile endurance and excitability, irrespective of the global fiber [ATP].

Direct in-vivo or in-situ Na^+^/K^+^ fluxes and ATP hydrolysis rate measures to determine NKA activity are difficult to obtain and are mostly done before or after muscle activity (see concurrent review by (McKenna et al. 2023). Alternatively NKA function can be estimated indirectly in an in vivo setting by estimating the muscle fiber membrane’s ability to respond to two closely spaced AP and hence the repriming time, define as the time interval for the second pulse to generates an AP, depends in part on NKA activation. With this measure of NKA activity, a depolarization of the t-system increases the repriming time as expected, however, the addition of phosphoenolpyruvate, which increases glycolytic ATP resynthesis, decreases the repriming period (Dutka and Lamb 2007a, b). When glycogen is enzymatically lowered with glucoamylase treatment, the repriming period increases (Watanabe and Wada 2019). The role of glycogenolytically-derived ATP was studied by the use of glycogen phosphorylase inhibitors and glycogen lowering treatment in mechanically skinned fibers, which invariably prolonged repriming time, strongly indicating an attenuated NKA activity (Jensen et al. 2020), further supporting the concept of a direct role of glycogenolytically-derived ATP on NKA activity in skeletal muscle, irrespective of bulk [ATP].

In summary, substantial evidence indicates a tight coupling between metabolism, via the glycogenolytic-glycolytic-derived ATP production, and NKA in muscle. As a consequence of lower glycogen and lower derived ATP production, a subsequent attenuation of increases in NKA activity can then result in greater K^+^-induced loss of membrane excitability and force as discussed in the section above entitled “*Modulation of the K*^+^*-induced force depression by NKA*”.

### K_ATP_ channels: a second metabolism-excitability link

The regulation of the K_ATP_ channel is a second mechanism that links energy metabolism to membrane excitability. In this section, we briefly discuss the channel activation properties followed by another section on its physiological role. The molecular structure, regulation, pharmacological properties and physiological roles of K_ATP_ channels in various tissues are detailed in other reviews (Aguilar-Bryan and Bryan 1999; Inagaki et al. 1996; Babenko et al. 1998; Foster and Coetzee 2016; Seino 1999). In skeletal muscle, K_ATP_ channel is composed of two subunits: Kir6.2, a weak inward K^+^ rectifier with four ATP binding sites, and SUR2A, a regulatory subunit with ATP/ADP binding sites. ATP closes the channel in the µM range when it is bound to the Kir6.2 subunit (Barrett-Jolley et al. 1996; Noma 1983; Vivaudou et al. 1991). Thus, one expects that K_ATP_ channels are closed in resting muscle as ATP levels are about 5–9 mM (Greiner and Glonek 2021; Hargreaves and Spriet 2018). Indeed, in vitro studies demonstrated that K_ATP_ channels are closed under voltage clamp conditions with mM ATP levels on the sarcolemmal cytoplasmic side (Vivaudou et al. 1991). However, two studies using *in-situ* skeletal muscle preparations demonstrated that a small number of K_ATP_ channels are open at rest, because glibenclamide, a K_ATP_ channel antagonist, reduced [K^+^]_int_ in human muscle (Nielsen et al. 2003) and lowered K^+^ efflux to almost zero in perfused rabbit muscle (Lindinger et al. 2001). Thus, other factors must be modulating K_ATP_ channel activity in resting skeletal muscle under *in-situ* conditions, one possible factor being insulin (Tricarico et al. 1997, 1999).

Electrically stimulating mouse tibialis muscle at 1 Hz resulted in sarcolemmal hyperpolarization by 10 mV, reduction in AP overshoot by 14 mV, as well as reduction in SR Ca^2+^ release (Zhu et al. 2014). Zhu et al. (2014) further showed that none of these changes were observed in K_ATP_ channel deficient muscles; i.e., tibialis muscle from Kir6.2 knockout (Kir6.2^−/−^) mouse model. The authors concluded that 1 Hz stimulation resulted in an activation of K_ATP_ channels. Exposing skeletal muscles to ischemia or chemical metabolic inhibition causes large K_ATP_ channel activity (Gramolini and Renaud 1997; Castle and Haylett 1987; Allard et al. 1995; Pang et al. 1997). Importantly, K_ATP_ channels are activated during fatiguing contractions. Stimulating rat soleus muscle with 3.5-s AP trains at 15 Hz repeatedly every 7 s eventually caused a 14-fold increase in G_K_, which was blocked by glibenclamide, a K_ATP_ channel blocker. The authors suggested that the increased G_K_ occurred as metabolic stress triggers fatigue and was due to an increased K_ATP_ channel activity (Pedersen et al. 2009a). Furthermore, large excitability, contractile and metabolic dysfunctions as well as fiber damage occur in the absence of K_ATP_ channel activity during fatigue (see section entitled “*K*_*ATP*_* channels: another physiological function for K*^+^ *during fatigue*”). Together, these results strongly suggests that not only are K_ATP_ channels activated during fatigue but that they are crucial at protecting muscles from deleterious exhaustion of [ATP].

The mechanism by which K_ATP_ channels are activated during fatigue is still not well understood. Firstly, the decreases in bulk [ATP] during or at the end of a fatigue bout range between 10 and 50% (Scott et al. 2016; Nagesser et al. 1993; Meyer and Terjung 1979; Whitlock and Terjung 1987; Mainwood et al. 1972; Dawson et al. 1978); i.e., ATP does not fall to the µM range necessary to activate the channel, raising the question as to whether low ATP itself is the main activator of K_ATP_ channels during fatigue. However, similar to NKA, the ATP that blocks K_ATP_ channel is primarily provided by glycolysis, at least in cardiac muscle, and there is close physical association and functional interaction between glycolytic enzymes and K_ATP_ channels (Weiss and Lamp 1989; Hong et al. 2011; Dhar-Chowdhury et al. 2005). It is therefore possible that the sub-sarcolemmal [ATP] becomes much lower than the bulk [ATP] allowing an activation of K_ATP_ channels, especially when glycogen is low, as discussed above for NKA. There are also other metabolites that reduce the ATP inhibition of K_ATP_ channels; including an increase in ADP (Vivaudou et al. 1991), decrease in intracellular pH as observed during fatigue (Davies et al. 1992; Standen et al. 1992; Allard et al. 1995) and increases in extracellular adenosine via its A1 receptor (Barrett-Jolley et al. 1996).

Taken together these studies have long suggested that K_ATP_ channels link energy metabolism to membrane excitability. That is, decreases in [ATP] as well as other metabolite changes during any metabolic stress result in the ATP dissociation from the channel, and as the channel opens it lowers AP amplitude and membrane excitability to ultimately reduce Ca^2+^ release and force generated or work done by muscle. This may in turn prevent damaging ATP depletion, under conditions with a high ATP turnover.

### *K*_*ATP*_*channels: another physiological function for K*^*+*^*during fatigue*

To study the physiological role of K_ATP_ channels, one must take a similar approach to that used for studying the role of any proteins, which is to determine the physiological response during fatiguing stimulation while the channel is either activated or blocked. Activating K_ATP_ channels in mouse EDL and soleus muscles with pinacidil lowered AP overshoot in unfatigued muscle (Gong et al. 2003). Although there is evidence for K_ATP_ channel activation during fatigue (Pedersen et al. 2009a), pinacidil further increased, compared to control, K^+^ efflux as well as the rate at which M-wave area and tetanic force decreased in mouse EDL and soleus muscle when fatigue was triggered with one tetanic contraction every sec for 3 min (Gong et al. 2003; Matar et al. 2000). Pinacidil had none of these effects in Kir6.2^−/−^ muscles, which do not express functional K_ATP_ channels. This suggests that not all K_ATP_ channels are activated during fatigue in control conditions and that the mechanism of action of the K_ATP_ channel involves an increased outward K^+^ current, which counteracts the Na^+^ inward current resulting in less depolarization and smaller AP in resting muscles and during fatigue. Furthermore, decreases in AP amplitude has been shown to reduce the SR Ca^2+^ release (Zhu et al. 2014; Wang et al. 2022). Considering the activation of K_ATP_ channels during fatigue (Pedersen et al. 2009a) and its link to metabolic state, it is more than likely that one essential function of the channel is to lower membrane excitability in order to prevent damaging ATP depletion by reducing Ca^2+^ release and force so that ATP utilization by SR Ca^2+^- and myosin- ATPases are reduced when ATP production is no longer sufficient to meet the demand.

Accordingly, one should expect that the absence of K_ATP_ channel activity leads to an impairment of muscle function. To test this, two approaches has been used to abolish K_ATP_ channel activity in skeletal muscle: (i) pharmacologically exposing wild type muscles to glibenclamide, a K_ATP_ channel blocker, and (ii) genetically using a knockout model in which the Kir6.2 subunit is no longer expressed; i.e., muscles from Kir6.2^−/−^ mice. Notably, in all the studies described here, the effects of abolishing K_ATP_ channel activity were quantitatively the same for the pharmacological and genetical approaches. Furthermore, glibenclamide had no effect in Kir6.2^−/−^ skeletal muscles (Cifelli et al. 2007). These results suggest that the different physiological responses between normal and K_ATP_ channel deficient muscles were due to a lack of channel activity and neither to some non-specific glibenclamide effect nor to other effects associated with a lack Kir6.2 expression. Experiments in vitro were initially carried out with mouse EDL and soleus with a fatigue protocol consisting of one 200 ms long tetanic contractions every s for 3 min (Matar et al. 2000; Gong et al. 2000). The major impact of blocking K_ATP_ channels were slightly faster decrease in tetanic force, greater increase in unstimulated force, defined as the force between contractions, and a reduced capacity to recover force following fatigue.

Experiments were then repeated using smaller muscle preparations, i.e., FDB muscle bundles and single fibers (Cifelli et al. 2008, 2007; Selvin and Renaud 2015). Compared to wild type FDB muscles, the lack of K_ATP_ channel activity led to four major impairments during fatiguing contractions. (1) The decreases in active [Ca^2+^]_i_ and tetanic force were faster and the final extent of the decrease greater. (2) The increases in unstimulated [Ca^2+^]_i_ and force, measured between contractions, were greater. In some cases the increase in unstimulated [Ca^2+^]_i_ was so large that unattached single fibers supercontracted from an elongated fiber to a very small square structure. (3) Resting E_M_ of several fibers depolarized to greater extent from a mean −80 mV to −30 mV (compared to just −60 mV for control conditions). Large depolarizations during fatigue were also reported in rat EDL exposed to glibenclamide (Pedersen et al. 2009b). (4) The capacity to recover tetanic force following fatigue was largely reduced. The authors suggested that the absence of K_ATP_ channel activity first lead to an excitability impairment as the large depolarization results in greater Na_V_1.4 channel inactivation compared to control contributing to greater decreases in AP amplitude, active [Ca^2+^]_i_ and tetanic force. The depolarization was also large enough to cause the opening of t-tubular Ca_V_1.1 channels allowing for large Ca^2+^ release between contractions. This was confirmed by exposing FDB to 0.6 mM Ca^2+^ (vs. 2.3 mM) or to 1 µM verapamil, a Ca_V_1.1 channel blocker at a concentration that had no effect on the pre-fatigue tetanic contraction of Kir6.2^−/−^ FDB. Both lower [Ca^2+^]_e_ and verapamil significantly reduced during fatigue the rate at which tetanic force decreased and the extent of the increase in unstimulated force, while it fully restored the capacity to recover force following fatigue in K_ATP_ channel deficient muscle. Based on these results, the authors suggested that the contractile impairment in regard to tetanic force during fatigue and recovery in K_ATP_ channel deficient FDB were in part due to some cellular damages possibly caused by the high unstimulated [Ca^2+^]_i_.

Notably, experiments with treadmill running further confirmed the lower fatigue resistance and the appearance of fiber damage in active Kir6.2^−/−^ muscles (Thabet et al. 2005). In regard to lower fatigue resistance, Kir6.2^−/−^ mice ran shorter distances when elicit to run on treadmill at 24 m/min with 20° inclination. On the first day of running wild type mice ran 2 km before they could no longer maintain the speed whereas Kir6.2^−/−^ mice only ran 0.5 km. After five consecutive running days, wild type mice had increased their running distance to 5 km while it only increased to 1.5 km for Kir6.2^−/−^ mice. In regard to fiber damage, 12% of plantaris and EDL muscle fibers of Kir6.2^−/−^ mice had centrally located nuclei, which occurs when a fiber had been damaged and regenerated by satellite cells; a value that was 25% in tibialis anterior. Less than 0.5% of fibers had central nuclei in the same muscles of wild type mice. Fiber damage occurred primarily in type IIB Kir6.2^−/−^ fibers. Finally, severe fiber damage with no evidence of fiber regeneration was also reported for Kir6.2^−/−^ diaphragm.

A last series of experiments demonstrated several metabolic dysfunctions in Kir6.2^−/−^ compared to wild type FDB (Scott et al. 2016). (1) ATP content decreased by about 5 µmoles/g wet weight during the first 30 s of fatigue in both wild type and Kir6.2^−/−^ FDB bundles. Thereafter, it slowly returned back to pre-fatigue levels by the second min of the 3 min fatigue bout in wild type but not in Kir6.2^−/−^ FDB. (2) At the end of the fatigue bout, there was a net loss of total adenylates (ATP + ADP + AMP contents). (3) Compared to wild type, Kir6.2^−/−^ FDB had greater glucose uptake, similar glycogen mobilization and greater glucose oxidation during the first min that resulted in a 3.5-fold greater ATP production. (4) However, during the remaining 2 min of the fatigue period glycogen was no longer mobilized and oxidative phosphorylation stopped. The decrease in ATP production and the large increases in unstimulated [Ca^2+^]_i_ and force between contractions increases the ATP demand by Ca^2+^ and myosin ATPases, which most likely were the cause for the lack of increase in ATP content in Kir6.2^−/−^ FDB that occurred in wild type FDB during the final two min of the fatigue bout.

In summary, K_ATP_ channels are activated during fatigue. They contribute to the reduction of AP amplitude, which then lowers SR Ca^2+^ release and force to preserve ATP as it reduces the ATP demand by Ca^2+^ ATPase and myosin ATPase. The lack of K_ATP_ channel activity during fatigue causes: (i) fiber damage and (ii) major excitability, contractility and metabolic dysfunctions suggesting that the channel is crucial in terms of myoprotection. That is, once activated, the channel prevents massive sarcolemmal depolarization and the subsequent large increases in unstimulated [Ca^2+^]_i_ and force. The apparent faster fatigue rate and lower force recovery in K_ATP_ channel deficient muscles, using either glibenclamide in wild type muscles or Kir6.2^−/−^ muscles, is most likely because of (i) Na_V_ channel inactivation associated with the large depolarization as well as (ii) fiber damage associated with greater increase in unstimulated [Ca^2+^]_i_ and ATP depletion.

### ***CLC-1 CL***^***−***^*** channel: a third potential metabolism-excitability link***

As discussed above in the section entitled “*Modulation of the K*^+^*-induced force depression by changes in G*_*Cl*_”, changes in ClC-1 channel activity or G_Cl_ significantly alter how changes in [K^+^]_e_ affect membrane excitability and force in resting, unfatigued muscles. For these observations to be of any physiological significance, one then expects changes in G_Cl_ during muscle activity and fatigue. In fact, there is evidence for the activation of ClC-1 channels during metabolic stress such as metabolic inhibition and fatigue (Fink and Luttgau 1976; Pedersen et al. 2009a, 2009b). In other words, ClC-1 channels may be a third membrane component for which its activity is regulated in part by the fiber metabolic status.

In two studies (Pedersen et al. 2009b, 2009a), 3.5 s long trains of APs at 15 Hz were triggered every seven seconds in rat/mouse EDL and rat soleus muscle fibers. At the onset of stimulation, G_Cl_ decreased by ~ 70% due to phosphorylation of ClC-1 channels by PKC while G_K_ increased very slightly; this period was called phase 1. Notably, there is no sign of any decreases in AP amplitude during phase 1. In rat EDL muscle, both G_Cl_ and G_K_ increased drastically after almost 2000 APs, this period was called phase 2. The increase in G_Cl_ was ~ threefold above pre-stimulation levels while the increase in G_K_ was ~ 14-fold and was entirely due to activation of K_ATP_ channels. Two other studies then reported similar phases 1 and 2 in mouse EDL and human abdominal and intercostal skeletal muscles; the decrease in G_Cl_ during phase 1 in human muscles was also due to ClC-1 channel phosphorylation by PKC (Riisager et al. 2014, 2016). Pedersen et al. (2009a) suggested that phase 2 was associated with substantial reduction in muscle fiber energetic state, based on the following evidence.

First, metabolic poisoning results in substantial increases in both G_Cl_ and G_K_, the latter being related to an activation of K_ATP_ channels (Fink and Luttgau 1976; Gramolini and Renaud 1997; Allard et al. 1995). Second, ClC-1 channels are ATP sensitive. Decrease in [ATP] shifts the steady state activation kinetics of ClC-1 channels toward more negative resting E_M_ (Tseng et al. 2007, 2011; Zhang et al. 2008); i.e., the shift allows for greater ClC-1 activity at a given resting E_M_. Third, that both ClC-1 and K_ATP_ channel activity increase simultaneously during phase 2, strongly supports the concept that this phase occurs when there is a metabolic stress. Fourth, contrary to rat EDL muscle, a glycolytic and fatigable muscle, in rat soleus, an oxidative and fatigue resistant muscle, phase 2 was not observed even after 15,000 APs (Pedersen et al. 2009b). Fifth, in EDL muscle phase 2 is triggered sooner and G_M_ increases to a greater extent in the absence than in the presence of glucose in the extracellular milieu (Pedersen et al. 2009b).

Three major facts can be made from all the studies discussed so far. First, it has long been known that large changes in plasma, interstitial and intracellular concentrations of K^+^ and Na^+^ observed during fatigue have the capability to affect sarcolemmal excitability and thus SR Ca^2+^ release and force/work in skeletal muscle. Second, observations of both the K^+^-induced force potentiation and depression appeared early last century in the literature. However, K^+^-induced potentiation has been much less prominently featured, often receiving only brief mention in reviews concerning the role of K^+^ effects in fatigue (Sejersted and Sjøgaard 2000; Cairns and Lindinger 2008; McKenna et al. 2008) while in two reviews a K^+^ role in muscle fatigue was questioned (Sjøgaard 1991; Allen et al. 2008b). Third, there is now evidence for a link between sarcolemmal excitability and muscle energy status involving at least three sarcolemmal components: NKA, K_ATP_ and ClC-1 channels.

## A new perspective for the role of K^+^, Na^+^ and Cl^−^ on muscle performance from the onset of exercise to fatigue

The documented contrasting effects of K^+^ on contractile performance begs the question as to when [K^+^]_e_ is a positive or negative modulator of performance during stimulated muscle contractions or during exercise in humans. This question has been discussed in at least two previous reviews (Renaud 2002; McKenna et al. 2008). Renaud (2002) emphasized that K^+^ has in fact several physiological roles during muscle activity and first proposed that K^+^ optimizes muscle performance at the onset of muscle activity and in the absence of metabolic stress. That is, K^+^ (i) potentiates twitch and sub-tetanic force, (ii) augments blood flow by triggering blood vessel vasodilation (Wilson et al. 1994; Knot et al. 1996; Armstrong et al. 2007), and (iii) activates muscle pressor reflex that increases heart rate and arterial blood pressure (MacLean et al. 2001; Rybicki et al. 1984) possibly via an activation of muscle metabosensitive afferent fibers (Laurin et al. 2010). Further details about how increases in plasma [K^+^] affects the respiratory and cardiovascular function can be found elsewhere (Paterson 1996). Then, when a metabolic stress occurs as ATP demand exceeds ATP supply, fatigue is triggered in which the K^+^ effect switches from force potentiation to depression in order to reduce ATP utilization and prevent potential damaging ATP depletion. At the time, some of the factors that modulate the K^+^ effects on force were unknown. With the knowledge of how changes in G_Cl_ modulate the K^+^ effects and how it changes during muscle activity we now further elaborate the concept first proposed by Renaud (2002). Three factors are taken into consideration: (i) [K^+^]_e_ itself, (ii) stimulation frequency and iii) muscle physiological/metabolic state as regulators of NKA activity, G_Cl_ (ClC-1 channels) and G_K_ (K_ATP_ channels) during exercise and fatigue.

### ***[K***^+^***]***_***e***_*** and stimulation frequency***

**Fig. 2:**
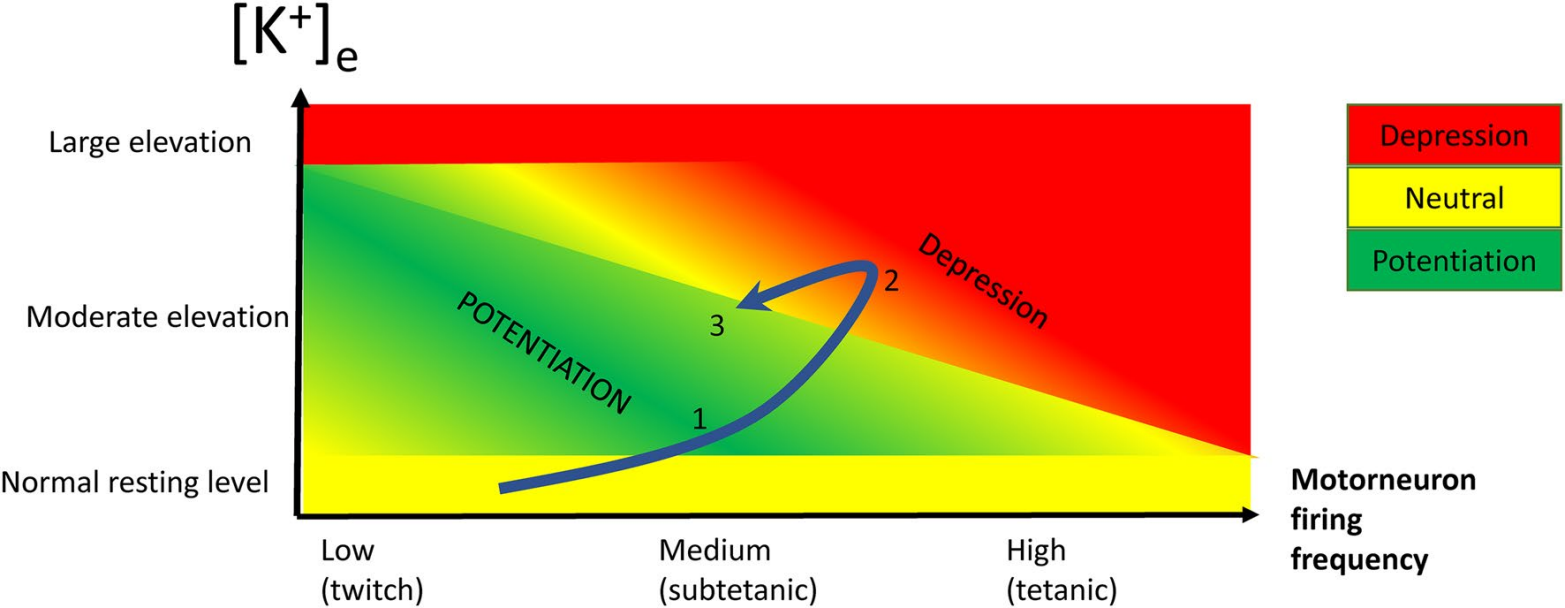
Effect of [K^+^]_e_ and motorneuron firing frequency on contractile performance: a balance between positive and negative effects of muscle [K^+^]_e_. Diagram depicting the proposed effects of [K^+^]_e_ on muscle force at various levels of [K^+^]_e_ increase and at various activation frequencies. The colors indicate potentiation (green) or depression (red) of force. The arrow and numbered points refer to various situations during exercise as explained in the text. Note that a given moderate elevation of [K^+^]_e_ can give rise to either potentiation or depression of force depending on the motorneuron firing frequency

The first two factors are interrelated as *in-vivo* stimulating frequencies directly affect increases in [K^+^]_e_. Considering the range of known activation frequencies (Hennig and Lomo 1985; Enoka and Fuglevand 2001) and the observed [K^+^]_i_ and [K^+^]_int_ during both maximal and submaximal exercise (McKenna et al. 2023), it is conceivable that K^+^ may exert both positive and negative effects depending on the exercise intensity and duration, and may even be involved in regulating exercise intensity by exerting these effects within a given exercise session. These impacts are conceptualized in Fig. 2. Consider an individual who embarks on a run at an initial slow pace. Here, muscle [K^+^]_int_ will increase to a moderate degree and motor units will be activated mainly with low or moderate activation frequencies, rendering the influence of K^+^ positive, due to its location on the [K^+^]_e_ –motorneuron firing frequency continuum (point “1” in Fig. 2). Increasing speed of running will require a higher motorneuron firing frequency, leading to further increase in [K^+^]_int_. If [K^+^]_int_ is sufficiently high to move into the red zone (point “2” in Fig. 2) then fatigue inevitably ensues. However, it is known that during fatiguing contractions, the motor unit motorneuron firing frequency diminishes (Bigland-Ritchie et al. 1983). Furthermore, such reduction in stimulation frequencies allows for a decreased loss or even recovery of force (Jones et al. 1979), partially because it allows more time for the membrane to repolarize between APs and partially because it reduces cellular K^+^ loss and subsequently [K^+^]_int_ with the end result of rendering the K^+^ effects to potentiating again (point “3” in Fig. 2). Furthermore, as exercise continues, muscle [K^+^]_int_ can actually decline from an early peak of > 10 mM even with unchanged exercise intensity (Nielsen et al. 2004a), presumably due to factors such as increased muscle NKA activity, elevated blood flow, increased oxidative energy supply and possibly reduced motor unit motorneuron firing frequency. Thus, the influence of elevated [K^+^]_e_ on contractile performance may be continuously fluctuating between potentiation and fatigue effects and thereby both aid initial or submaximal exercise performance, but also help determine the limits for muscular performance during intense activities, in a regulated balance involving motor output from the central nervous system as well as various hormonal factors (catecholamines and CGRP) that modulate the K^+^ effect. Nevertheless, it should be noted that during non-fatiguing moderate exercise, where activation frequencies are sub-maximum and increased [K^+^]_e_ are low/moderate, it seems reasonable to consider K^+^ to be a primarily positive regulator of contractile performance in muscles.

### Metabolic state sarcolemmal excitability link: role of NKA, K_ATP_ and CLC-1 channel

**Fig. 3:**
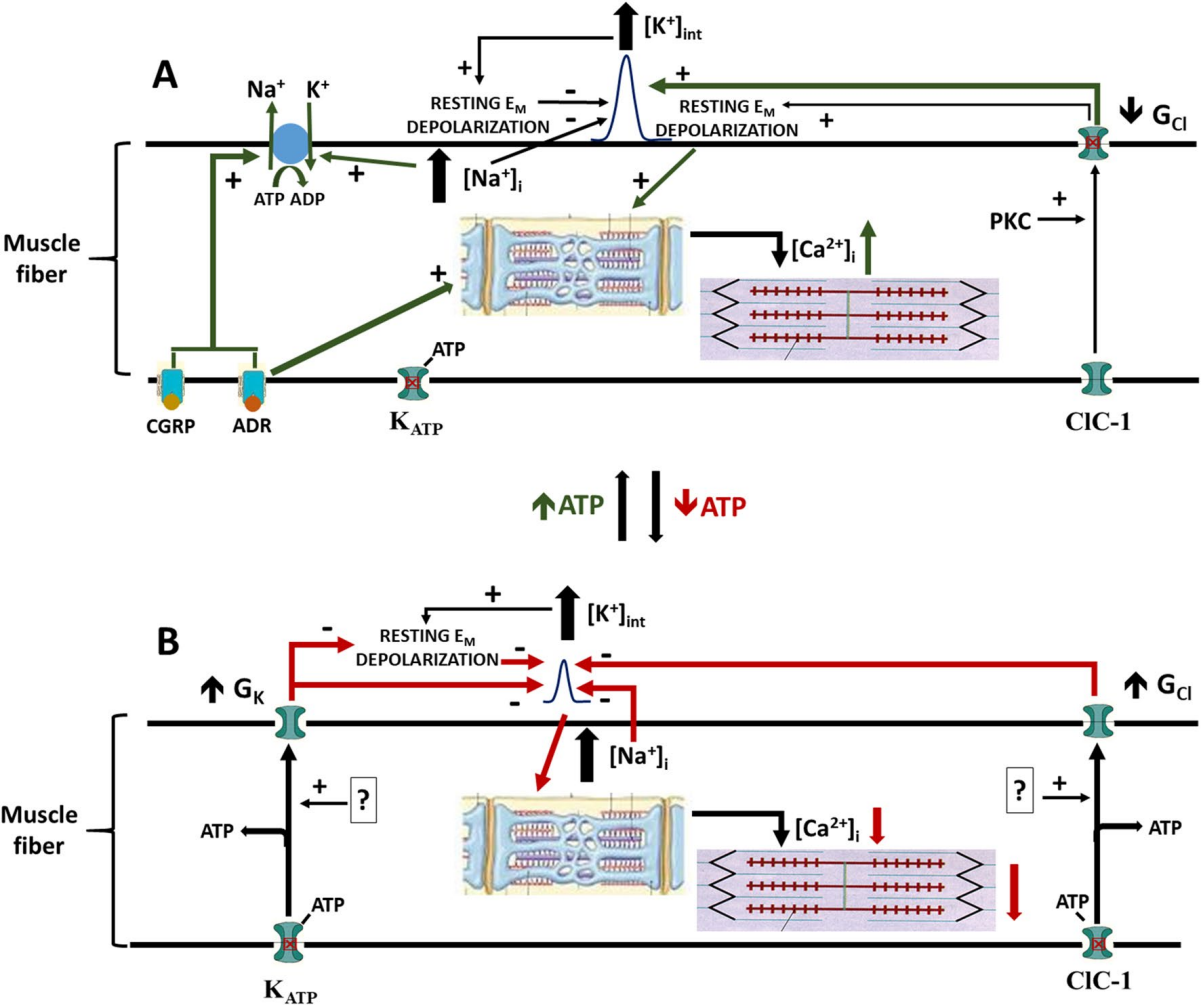
A modified concept for the physiological roles of K^+^ in combination with combined effects of Na^+^, Cl^−^, NKA activity, and ClC-1 and K_ATP_ channels, during muscle activity in the absence and presence (i.e., fatigue) of metabolic stress. **A** In the absence of metabolic stress, the expected high [K^+^]_int_- and [Na^+^]_i_-induced loss of membrane excitability due to increases in [Na^+^]_i_ and [K^+^] _int_ and thus force is counteracted by the closure of ClC-1 Cl^−^ channels following phosphorylation by PKC and by an increased NKA activity due to its activation by catecholamines (via their adrenergic receptors, ADR), CGRP as well as elevation in [Na^+^]_i_. This occurs because under those conditions the critical [K^+^]_int_ that causes force depression is shifted to higher [K^+^]_int_, which also shifts the critical resting E_M_ to less negative potential. Furthermore, sub-maximal tetanic forces are potentiated by the increased [K^+^]_int_ as well as by catecholamines increasing SR Ca^2+^ release. **B** During metabolic stress, ClC-1 and K_ATP_ channels are activated in part by the decrease in ATP concentration allowing ATP dissociation from the channels and in part by intracellular signaling pathway(s) that has yet to be identified. The increases in ClC-1 channel activity (G_Cl_) and in K_ATP_ channel activity (G_K_) directly reduce sarcolemmal excitability by providing an outward Cl^−^ and K^+^ currents that (i) move the action potential threshold toward less negative E_M_ and (ii) counteract the Na^+^ inward current during the depolarization, thereby reducing action potential amplitude. The increase in G_Cl_ also lowers the critical [K^+^]_e_ switching the K^+^ effect from force potentiation to depression

At the onset of any muscle activity, one expects that ATP demands are met by adequate ATP production so that there is no metabolic stress related to an energy deficit. Four major events occur under this metabolic/energy condition (Fig. 3A); (i) large increases in [K^+^]_int_ that can reach 10–12 mM as observed even in moderate 30 W knee extension exercise (Nielsen et al. 2004a), (ii) increases in [Na^+^]_i_, (iii) increases in NKA activity due to increases in [Na^+^]_i_, activation by catecholamines and CGRP, and iv) decreases in G_Cl_ as ClC-1 channels close following phosphorylation by PKC (as observed during phase 1 in the Pedersen et al. studies (2009). The expected high [K^+^]_int_- and [Na^+^]_i_-induced loss of membrane excitability and thus force as discussed above is counteracted by the increased in NKA activity and closure of ClC-1 Cl^−^ channels. This occurs because under those conditions the critical [K^+^]_e_ that causes force depression is shifted to higher [K^+^]_e_, which also shifts the critical resting E_M_ to less negative potential. Furthermore, sub-maximal tetanic forces are potentiated by the increased [K^+^]_int_ as well as by catecholamines increasing SR Ca^2+^ release. The duration of this condition (i.e., phase 1) lasts as long as there is no or minimal metabolic stress becoming shorter as the muscular activity becomes more intense.

When muscle ATP does not meet the demand, especially in restricted areas of the muscle fiber, the ATP depletion triggers phase 2. Phase 2 involves large increases in G_Cl_ and G_K_ as ClC-1 and K_ATP_ channels are activated in part by the decrease in ATP concentration allowing the ATP dissociation from the channels and in part by intracellular signaling pathway(s) that has yet to be identified (Fig. 3B). The increases in ClC-1 channel activity or G_Cl_ and in K_ATP_ channel activity or G_K_ directly reduce sarcolemmal excitability by providing an outward Cl^−^ and K^+^ outward currents that (i) move the action potential threshold toward less negative E_M_ and (ii) counteract the Na^+^ inward current during the depolarization reducing action potential amplitude. Although the effect of an increase in G_Cl_ on membrane excitability and force generation has not been studied directly due to the lack of a ClC-1 channel opener, one can expect that the increased G_Cl_ lowers the critical [K^+^]_e_ switching the K^+^ effect from potentiation to depression.

These physiological responses during phase 2 are critical to prevent damaging ATP depletion as well as excitability, contractile and metabolic dysfunctions that occur during fatigue in K_ATP_ channel deficient muscles, as discussed above in the section entitled “*K*_*ATP*_* channels: another physiological function for K*^+^ *during fatigue*”. Finally, as recently reviewed, after intense muscle activity plasma [K^+^] falls rapidly and can decline to below pre-exercise levels indicating ongoing elevated muscle NKA activity. (McKenna et al. 2023). This suggests that local and circulating factors (e.g., CGRP and catecholamines, respectively) that augment NKA activity during exercise may thereby also contribute to preventing excessive increases in muscle [K^+^]_int_ and [Na^+^]_i_ that might otherwise completely paralyze the contracting muscle.

The model proposed in Fig. 3 is based mostly on results obtained from animal studies. So, one can raise the issue as to whether it applies to human skeletal muscle. On the one hand, considering i) that a few studies reported similar changes in [K^+^]_i_, [Na^+^]_int_ and [Na^+^]_i_ between animal and human muscles, together with the larger number of reports on human muscle [K^+^]_int_ (Tables 1 and 3), one can expect that the model in Fig. 3 also applies to human muscles. Furthermore, the changes in G_Cl_ (i.e., phases 1 and 2) during repetitive AP firing that have been reported in rat and mouse EDL also occur in human skeletal muscles (Riisager et al. 2014, 2016; Pedersen et al. 2009b, 2009a). On the other hand, a decreased sarcolemmal excitability in human muscle during fatigue is controversial because most studies reported no change or increases in M-wave amplitude during fatigue in human muscles as previously reviewed (Allen et al. 2008b). Interestingly, it has recently been proposed that increases in M-waves amplitude are in fact evidence of excitability disruption, especially AP propagation (Rodriguez-Falces and Place 2018). This is because the M-wave has two components: (i) a propagating positive signal from AP and (ii) a non-propagating negative signal from the AP termination at the tendon. They discuss the possibility that under normal unfatigued conditions, the non-propagating negative signal is close to the propagating positive signal with the net effect of reducing the amplitude of the former. As fatigue occurs and as AP propagation slows down, the distance between the two signals increases resulting in less counteraction of the negative signal, resulting in greater amplitude of the positive signal. Thus, future studies will be necessary to better understand the significance of the M-wave signals and to determine if the model in Fig. 3 also applies to human muscles.

## Conclusions

K^+^ disturbances in muscle have long been considered a factor in the mechanism by which force/work decreases during fatigue in skeletal muscle. Studies have questioned this potential role based on (i) the increase in muscle [K^+^]_e_ during fatigue not being high enough by itself to induce force depression during fatigue, or actually declining during continued exercise and ii) the fact that for submaximal tetanic contraction, small increases in [K^+^]_e_ actually potentiate muscle force. This review emphasizes that K^+^ exerts dual roles. A first role is one of force potentiation during low-to-moderate exercise intensities, as part of several mechanisms that optimize muscle contraction. A second role is one of force depression that occurs during a metabolic stress that restricts ATP availability. At the onset of muscle activity and during moderate muscle activity, the K^+^-induced potentiation predominates while the K^+^-induced depression is prevented, primarily because of NKA activation and a reduced G_Cl_ as ClC-1 channels close. The K^+^-induced force depression in muscle occurs when metabolic stress/energy deficit occurs and leads to the activation of ClC-1 and K_ATP_ channels, which reduces sarcolemmal excitability and thus force. The K^+^-induced force depression is further enhanced by the synergistic depressive effect of reduced Na^+^ and K^+^ gradients.

## Future studies

Although the decrease in G_Cl_ in phase 1 involves ClC-1 channel phosphorylation by PKC, the mechanism by which metabolic stress activates both ClC-1 and K_ATP_ channels remains to be elucidated. Although ATP modulates the activity of both channels, where decreases in ATP result in greater channel activity, it is more than likely that intracellular signaling pathways are implicated. For example, the adenosine A1-receptor activates K_ATP_ channels under patch clamp conditions (Barrett-Jolley et al. 1996) while AMP kinase (AMPK), a well-known intracellular cell energy sensor, activates cardiac K_ATP_ channels during metabolic stress (Yoshida et al. 2012). Furthermore, it is important to bear in mind that the majority of mechanistic studies on muscle ion regulation and excitability are conducted in rodent muscles, with few studies in humans. Also, studies measuring compound action potentials (EMG) inherently only estimate muscle surface AP propagation and not t-tubule excitability, representing the larger part of the muscle membrane. In fact, little is known about t-tubule excitability even in rodent muscle. Thus, a better understanding of the importance of the t-system excitability in determining the force response under physiologically relevant conditions in human as well as animal skeletal muscles is requisite.

## Data Availability

All data discussed in this review comes from published work. There was no new data for this review.

## Abbreviations

9-AC: 9-Anthracene carboxylic acid
[Cl^−^]_e_: Extracellular [Cl^−^]
[K^+^]_e_: Extracellular [K^+^]
[Na^+^]_e_: Extracellular [Na^+^]
[Ca^2+^]_i_: Intracellular [Ca^2+^]
[K^+^]_int_: Interstitial [K^+^]
[Na^+^]_int_: Interstitial [Na^+^]
[Cl^−^]_i_: Intracellular [Cl^−^]
[K^+^]_i_: Intracellular [K^+^]
[Na^+^]_i_: Intracellular [Na^+^]
ADP: Adenosine diphosphate
AMP: Adenosine monophosphate
AMPK: AMP kinase
AP: Action potential
ADR: Adrenergic receptor
ATP: Adenosine triphosphate
Ca_V_1.1: Voltage-sensitive Ca^2+^ channel 1.1
CGRP: Calcitonin gene related peptide
ClC-1: Cl^−^ channel 1
E_Cl_: Cl^−^ equilibrium potential
E-C-R: Excitation–contraction-relaxation
EDL: Extensor digitorum longus
E_K_: K^+^ equilibrium potential
E_M_: Membrane potential
EMG: Electromyogram
FDB: Flexor digitorum brevis
HEK-293: Human embryonic kidney 293
Hz: Hertz
G_Cl_: Cl^−^ conductance
G_K_: K^+^ conductance
G_Na_: Na^+^ conductance
IMP: Inosine monophosphate
K_ATP_ channel: ATP-sensitive K^+^ channel
Kir6.2: Inward K^+^ rectifier 6.2
Kir6.2^−^^/^^−^: Knockout for the K_ATP_ channel subunit Kir6.2
K_V_: Voltage-sensitive K^+^ channel
mM: Millimolar
ms: Millisecond
mV: Millivolt
Na_V_: Voltage-sensitive Na^+^ channel
Na_V_1.4: Voltage-sensitive Na^+^ channel 1.4
NKA: Na^+^,K^+^-ATPase, Na^+^,K^+^-pump
PCr: Phosphocreatine
PKC: Protein kinase C
pH_e_: Extracellular pH
pH_i_: Intracellular pH
Resting E_M_: Resting membrane potential
SR: Sarcoplasmic reticulum
TPS: Train per s
t-tubule: Transverse tubule
TTX: Tetrodotoxin
W: Watt
µM: Micromolar
